# Transcription factor binding process is the primary driver of noise in gene expression

**DOI:** 10.1371/journal.pgen.1010535

**Published:** 2022-12-12

**Authors:** Lavisha Parab, Sampriti Pal, Riddhiman Dhar

**Affiliations:** 1 Department of Biotechnology, Indian Institute of Technology (IIT) Kharagpur, Kharagpur, West Bengal, India; 2 Max-Planck-Institute for Evolutionary Biology, Plön, Germany; University of Michigan, UNITED STATES

## Abstract

Noise in expression of individual genes gives rise to variations in activity of cellular pathways and generates heterogeneity in cellular phenotypes. Phenotypic heterogeneity has important implications for antibiotic persistence, mutation penetrance, cancer growth and therapy resistance. Specific molecular features such as the presence of the TATA box sequence and the promoter nucleosome occupancy have been associated with noise. However, the relative importance of these features in noise regulation is unclear and how well these features can predict noise has not yet been assessed. Here through an integrated statistical model of gene expression noise in yeast we found that the number of regulating transcription factors (TFs) of a gene was a key predictor of noise, whereas presence of the TATA box and the promoter nucleosome occupancy had poor predictive power. With an increase in the number of regulatory TFs, there was a rise in the number of cooperatively binding TFs. In addition, an increased number of regulatory TFs meant more overlaps in TF binding sites, resulting in competition between TFs for binding to the same region of the promoter. Through modeling of TF binding to promoter and application of stochastic simulations, we demonstrated that competition and cooperation among TFs could increase noise. Thus, our work uncovers a process of noise regulation that arises out of the dynamics of gene regulation and is not dependent on any specific transcription factor or specific promoter sequence.

## Author summary

Author summary

Expression levels of genes can vary even among genetically identical cells under identical environmental condition–a phenomenon termed expression noise. Gene expression noise has been experimentally measured in several cell populations and earlier studies have associated the presence of a specific sequence of bases such as the TATA box in the promoter region, the nucleosome occupancy levels and the histone modification patterns with high expression noise. However, how well these molecular features of a gene can let us predict its expression noise has not yet been assessed. In the current work, we test a large number of molecular features associated with gene expression for their ability to predict noise. We find that the number of transcription factors of a gene is a key predictor of expression noise. An increase in the number of transcription factors can change their binding process to the promoter region and can lead to more cooperation or competition. Through modeling and simulation of cooperative and competitive binding, we show that the transcription factor binding process primarily drives expression noise. Our work shows that the dynamics of gene expression regulation is the most important feature for predicting expression noise and uncovers a general mechanism of noise regulation.

## Introduction

Random fluctuations in molecular events occurring inside a cell generate variations in the expression levels of genes that is referred to as gene expression noise. Expression noise gives rise to variations in the activities of cellular pathways and generates phenotypic heterogeneity among individual cells of an isogenic population under identical environmental condition. Gene expression noise has important role in antibiotic persistence [1–5] and incomplete penetrance of mutations [6–10]. In addition, phenotypic heterogeneity has a key role in growth of cancers [11–13] and in emergence of therapy resistance [14–18].

Gene expression noise has been measured in some microbial systems [19–22] and its molecular origins have been widely investigated [23–36]. These studies have shown a correlation between presence of the TATA box motif in the promoter region of a gene and expression noise [20,26,29,37,38]. Further, promoter nucleosome occupancy, alone as well as in combination with presence of the TATA box motif, and histone modification patterns have also been associated with expression noise [33,35,39–43]. These features can influence transcriptional burst size and burst frequency [43–45] which in turn can impact expression noise [29,46–48]. However, even after so many studies over the years, the relative importance of these molecular features in noise regulation remains unknown. In addition, to what extent each of these molecular features can predict noise has not yet been quantified. That is, whether we can estimate the expression noise of a gene given the presence or absence of the TATA box sequence in its promoter and the promoter nucleosome occupancy pattern is not known. Thus, a predictive model of noise will be immensely helpful for a better understanding of noise regulation in biological systems.

In the current work, we report development of an integrated statistical model of gene expression noise in yeast by combining a large number of molecular features that can impact gene expression. We quantified the relative contribution of each of these features in explaining variations in noise values of genes and tested their predictive abilities. We observed that the presence of the TATA box and the promoter nucleosome occupancy pattern were poor predictors of expression noise. Instead, the number of regulatory TFs of a gene emerged as the key predictor of noise. An increase in the number of regulatory TFs was associated with a concomitant increase in the number of cooperative TFs. In addition, an increase in the number of regulatory TFs meant crowding of TF binding sites in the promoter region of a gene. This led to more overlaps between TF binding sites, thereby increasing competition between TFs for binding to the same promoter site. Mathematical modeling and stochastic simulations showed that a mere increase in the number of TFs could not explain the increase in expression noise, whereas cooperative and competitive TF binding could generate higher expression noise. Taken together, our work demonstrates that the binding process of transcription factors is the best predictor of noise in yeast. We uncover a dynamic noise regulation mechanism originating from competition and cooperation among transcription factors. This mechanism is not dependent on specific transcription factor or specific promoter sequence and thus, could be of interest to researchers working on different biological organisms.

## Results

### Quantification of expression noise at the level of mRNA and protein

**Fig 1:**
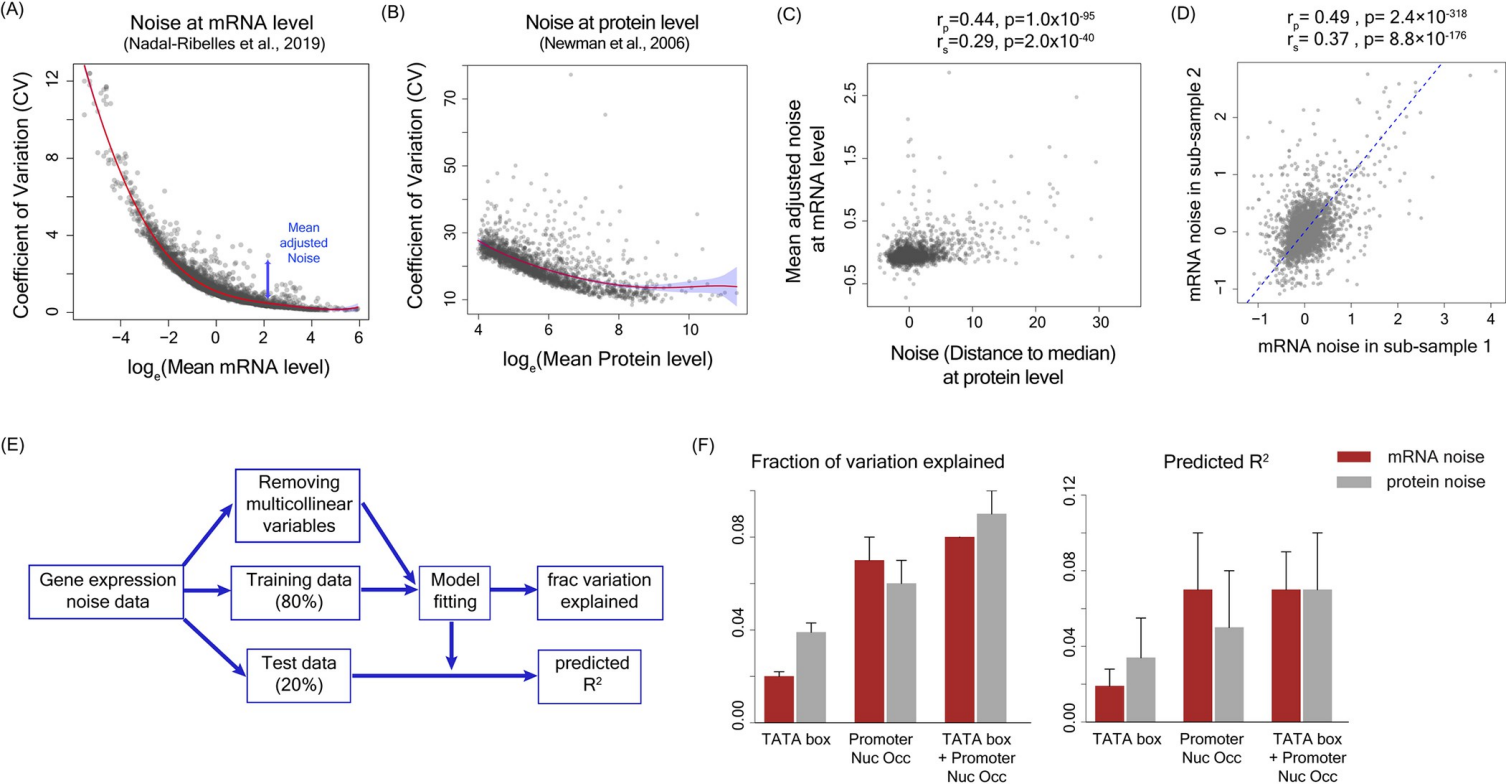
Presence of the TATAbox sequence and promoter nucleosome occupancy levels are poor predictors of gene expression noise. **(A)** Noise values calculated at the mRNA level from single cell RNA-seq data in yeast [49]. The mean adjusted noise was calculated by fitting a polynomial curve to the CV vs mean plot shown by the red line. Each point shows CV and mean mRNA level for a gene. **(B)** Noise values calculated at the protein level from flow cytometry measurements by [19]. The red line shows the best polynomial fit and the shaded blue region shows 95% confidence interval. **(C)** Correlation between mean-adjusted noise at the mRNA level and noise (DM) at the protein level. ‘r_p_’ shows Pearson’s correlation value and ‘r_s_’ shows Spearman’s correlation value. **(D)** Correlation of expression noise values at the mRNA level calculated from two sub-samples of the single-cell RNA-seq data [49]. **(E)** Flowchart showing the steps for model fitting, calculation of fraction of variation explained and derivation of predicted R^2^. **(F)** Fraction of variation explained and predicted R^2^ values by presence or absence of the TATA box sequence, average promoter nucleosome occupancy per nucleosome bound site and the combination of presence/absence of the TATA box sequence with promoter nucleosome occupancy.

We quantified gene expression noise at the level of both mRNA and protein using two different experimental datasets. For calculating noise at the level of mRNA, we used single-cell RNA-seq data in yeast from Nadal-Ribelles *et al*. [49] (Fig 1A). The dataset contained expression values of genes in 127 single-cells of *Saccharomyces cerevisiae* strain BY4741 grown in rich growth medium (YPD) and expression profiles measured at early-log phase. We obtained expression values of 5475 genes from this dataset. To quantify noise, we used a measure of noise that was independent of mean expression level through fitting a spline to the noise (coefficient of variation, CV) vs mean plot and calculating vertical distance of noise values from the fitted curve (Figs 1A and S1). Mean adjusted noise values from two sub-samples of the single-cell RNA-seq data showed significant correlation with each other (Pearson’s correlation r_p_ = 0.49, p = 2.4×10^−318^ and Spearman’s correlation r_s_ = 0.37, p = 8.8×10^−176^; Fig 1D).

We obtained noise values at the protein level for 2763 genes in *S*. *cerevisiae* S288C strain grown in rich medium (YPD) [19](Fig 1B). We used their measure of ‘distance to median’ (DM) as the measure of noise in our study (S1 Fig). Noise at the mRNA level showed significant correlation with noise at the protein level (r_p_ = 0.44, p = 1.7×10^−95^; r_s_ = 0.29, p = 2.0×10^−40^; Fig 1C) although the range of absolute noise values were very different. Genes showed a wide range of expression noise values with highly noisy genes showing large positive values and low-noise genes showing large negative values.

To quantify the relative importance of each molecular feature in noise regulation and to measure their ability to predict noise, we randomly segregated the noise data into training (80% of the full data) and test datasets (remaining 20%) (Fig 1E). For quantifying predictive ability of a single feature, we fitted a linear regression model to the training data at this step. For quantifying predictive ability of a combination of features, we first removed multi-collinear features and identified the key set of features through Ridge or Lasso regression on the full data and then fitted a linear regression model on the training data. This gave us the fraction of variation explained by the model (Fig 1E). We then used the fitted model to make predictions on the test data and computed predicted R^2^ values (Fig 1E). We performed this analysis in both mRNA and protein noise datasets to ensure that inferences drawn were not biased by a specific dataset.

Molecular features that had earlier been thought to impact expression noise, such as presence of the TATA box sequence in the promoter [20,29,37,38] and promoter nucleosome occupancy [39–41] showed significant association with noise (S2 Fig) but were poor predictors (Fig 1F). Specifically, the TATA box sequence, promoter nucleosome occupancy alone and in conjunction with the TATA box sequence could explain only ~2–4%, ~6–7% and ~8–9% of the noise variation, respectively and had low predictive power (predicted R^2^ value 0.02–0.03 for the TATA box alone, 0.05–0.07 for promoter nucleosome occupancy alone, and 0.07 for the TATA box + promoter nucleosome occupancy; Fig 1F). This suggested that these features were largely associated with noise and were not predictive.

### Molecular features associated with TF binding were the top predictors of noise

**Fig 2:**
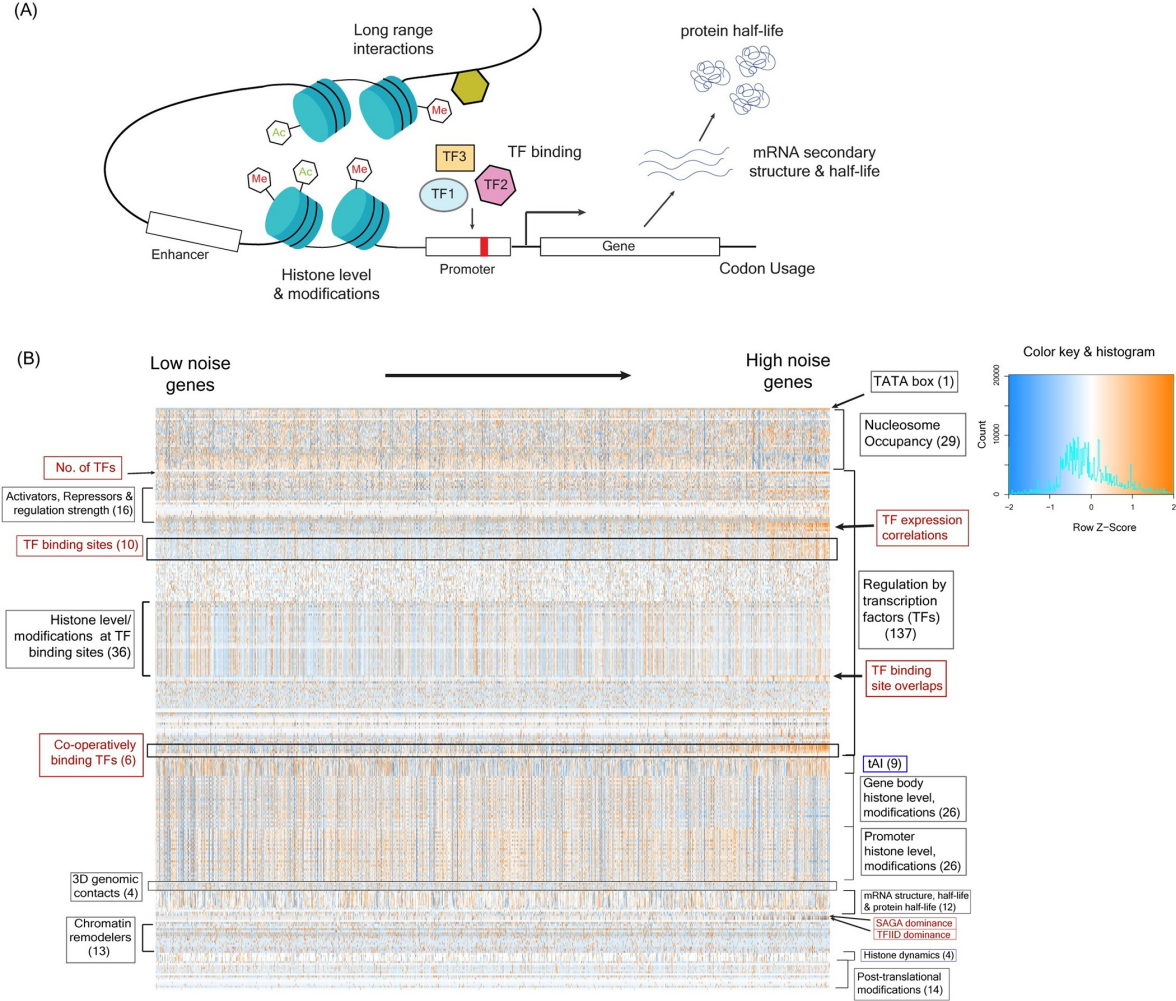
**An integrated statistical model of gene expression noise (A)** Schematic diagram depicting the molecular features that could impact gene expression and thus, could have a key role in regulation of expression noise. **(B)** An integrated model of noise constructed considering the TATA box sequence, absolute nucleosome occupancy levels, gene regulation by TFs, tRNA adaptation index, histone modification patterns in gene-body and promoter regions, 3D genomic contacts, mRNA structure and half-life, protein half-life, activity of chromatin remodelers, histone binding dynamics and post-translational modifications. The heatmap shows values of all these features (scaled and centered) in genes (represented in the columns) sorted according to their noise values at the protein level. The number of features for which the data is shown in the heatmap are indicated inside the brackets. Features highlighted in red appear different in their values between low and high noise genes. The panel on the right shows the color key for the heatmap along with the distribution of values of all features (histogram).

To identify molecular features that could explain the observed variations in noise values and could predict noise, we built an integrated statistical model considering a large number of features that were known or were likely to influence gene expression, as these could be potential regulators of noise (Fig 2 and S1 Table). The goals of the integrated statistical model were to test the predictive power of each molecular feature individually and to identify the best set of features for noise prediction out of a large number of possible combinations.

The molecular features incorporated in the integrated model included the number of regulating TFs, location of their binding sites, their mean expression and noise levels, SAGA/TFIID dependence of genes for their expression [50], whether a gene was co-activator redundant or TFIID dependent [51], binding activity of several broadly acting TFs such as TBP, ABF1 and RAP1 [52–54], binding patterns of chromatin remodelers [55–57], histone levels, histone modification patterns and histone binding dynamics [58,59], three-dimensional genomic contacts [60], tRNA adaptation index [61], mRNA secondary structure, mRNA and protein half-lives [62–64], post-translational modifications [65], in addition to nucleosome occupancy pattern [66] and presence/absence of the TATA box sequence in the promoter [67]. For a gene, we only considered those TFs for which experimental evidence for DNA binding had been obtained or change in expression upon knocking out the TF had been experimentally observed. For nucleosome occupancy, we not only considered the number of nucleosome-bound sites but included the absolute nucleosome occupancy pattern [66]. In total, we considered 329 features in our integrated model (See Methods, S1 Table).

**Fig 3:**
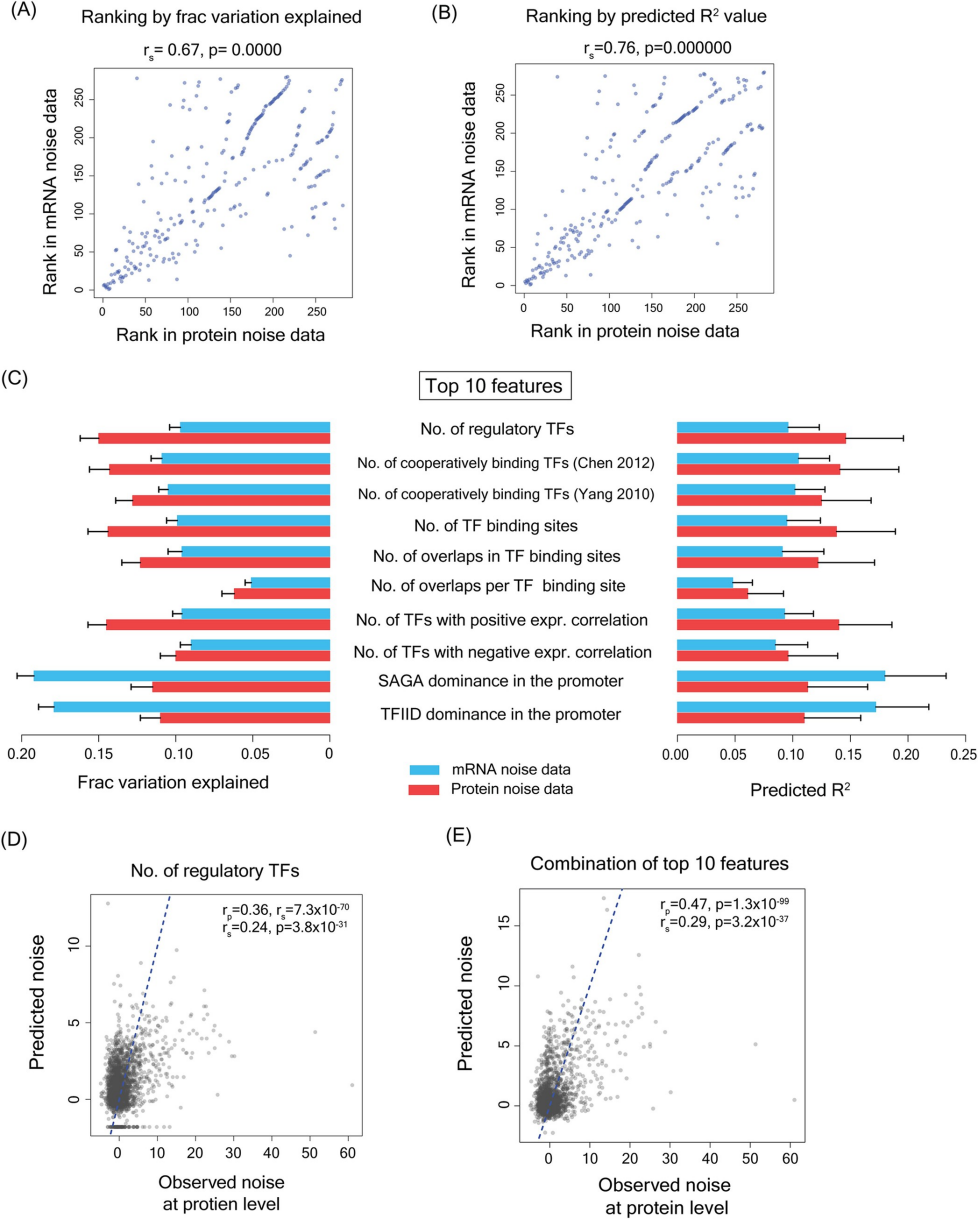
**Features with highest predictive powers were largely related to transcription factor binding process (A)** Rankings of features according to the fraction of variation explained in mRNA noise dataset and in protein noise dataset were highly correlated **(B)** Rankings of features according to the predicted R^2^ value in mRNA and protein noise datasets were highly correlated **(C)** Fraction of variation explained and predicted R^2^ value for top 10 features for both mRNA and protein noise datasets. **(D-E)** Correlation between observed noise values and noise values predicted by linear regression model considering a single feature (number of regulatory TFs) (D) and by the combination of top 10 features (E).

We tested each feature individually for its ability to explain variation in the noise data and to predict noise in both mRNA and protein noise datasets. We then ranked these features according to the fraction of variation explained and by predicted R^2^ values. The rankings of the features, whether based on the fraction of variation explained or the predicted R^2^ value were substantially correlated among mRNA and protein noise datasets with Spearman’s correlation values of 0.67 and 0.76, respectively (Fig 3A and 3B).

The top 10 features for explaining the variation existing in the noise data and for predicting noise values contained the same features although their rankings were slightly different. The distributions of values of some of these features are shown in S3 Fig. Interestingly, eight of these features were associated with TF binding, suggesting a key role for TFs in noise regulation (Fig 3C). These included number of regulatory TFs of a gene (fraction of variation explained ~0.1–0.15 and a predicted R^2^ of ~0.1–0.15) and the number of TF binding sites (fraction of variation explained ~0.1–0.14, predicted R^2^ ~0.1–0.14). Two features out of top 10 features were related to SAGA-dependence and TFIID-dependence of genes for their transcription. Stress response genes in yeast are known to be noisier than housekeeping genes [19]. While housekeeping genes are dependent on TFIID complex for their expression, stress response genes are usually SAGA complex dependent. SAGA dependence and TFIID dependence could explain 0.11–0.19 and 0.11–0.17 fraction of variation respectively with predicted R^2^ values of 0.11–0.18 and 0.11–0.17 respectively (Fig 3C).

We further validated predictive abilities of these features by correlating the observed and the predicted noise values. Predicted values obtained using number of regulatory TFs as the only feature and using the combination of top 10 features showed significant correlations with the observed noise values at the protein level (Fig 3D and 3E).

**Fig 4:**
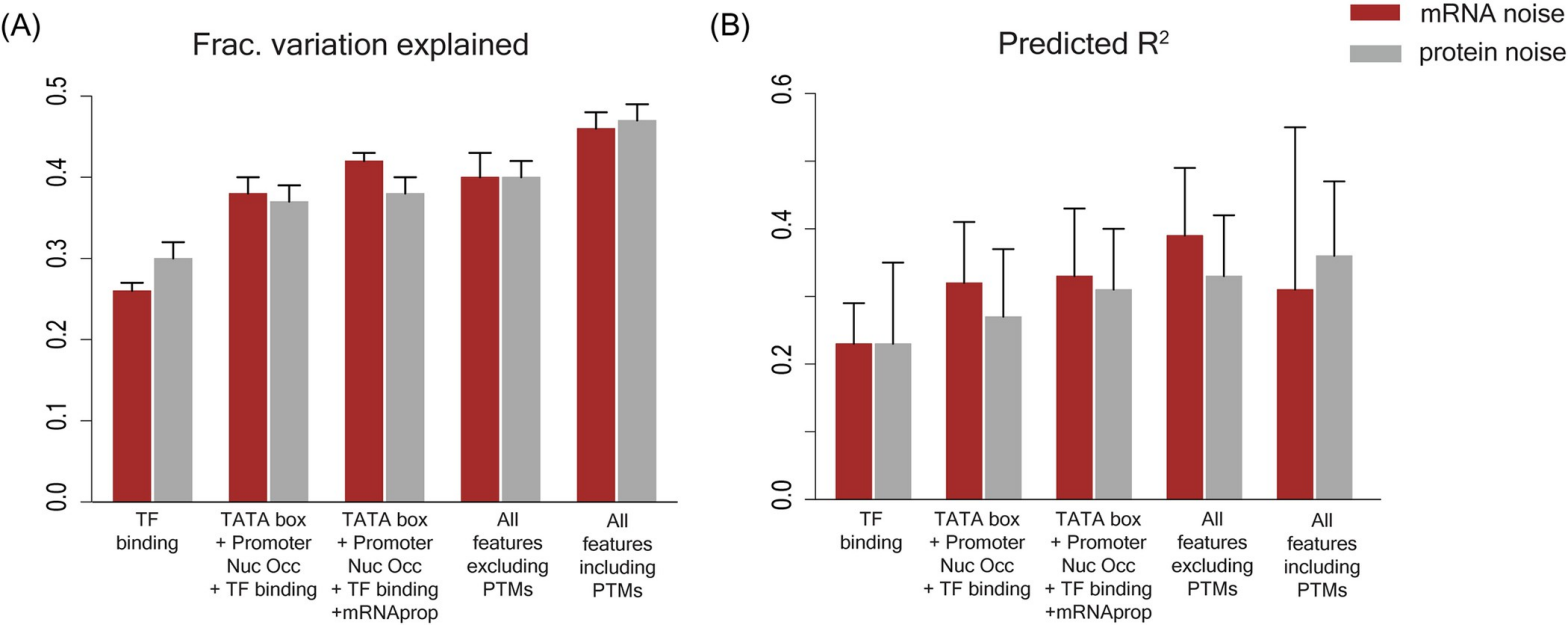
Fraction of variation explained and predictive ability of combinations of molecular features. **(A)** Fraction of variation explained in gene expression noise data and **(B)** predictive ability (given by predicted R^2^ value) by features associated with TF binding; combination of TF binding with the TATA box sequence and promoter nucleosome occupancy; combination of TF binding with the TATA box sequence, promoter nucleosome occupancy and mRNA properties; combination of all features excluding post-translational modifications (PTMs), and by combination of all features including PTMs.

Of all the features in our model, TF binding process could explain the largest part of the fraction of variation in the data and had the highest predictive power (Fig 4). The integrated model comprising of all features was able to explain 0.46 fraction of the variation in noise at the mRNA level and 0.47 fraction of the variation in noise at the protein level (Fig 4A). TF binding alone explained 0.26 fraction of the variation in the noise at the mRNA level and 0.30 fraction of the noise at the protein level (Fig 4A). In addition, the integrated model was able to predict noise at the mRNA level with predicted R^2^ value of 0.31 and at the protein level with predicted R^2^ value of 0.36 (Fig 4B). As before, TF binding process alone could predict noise at both mRNA and protein levels with predicted R^2^ value of 0.23 (Fig 4B).

Several genes in the yeast genome have been retained from a whole-genome duplication [68] and thus, share many molecular features including promoter and coding region sequences with their duplicates. This could bias our analysis and could lead to inflated predictive R^2^ values. Thus, to assess the impact of gene duplicates in our analysis, we removed duplicates from our datasets and repeated all analysis. The fraction of variation explained and predicted R^2^ values by individual features and by combinations of features were comparable between datasets with and without duplicate genes (S4 and S5 Figs).

### Genes with high expression noise were regulated by a higher number of TFs

**Fig 5:**
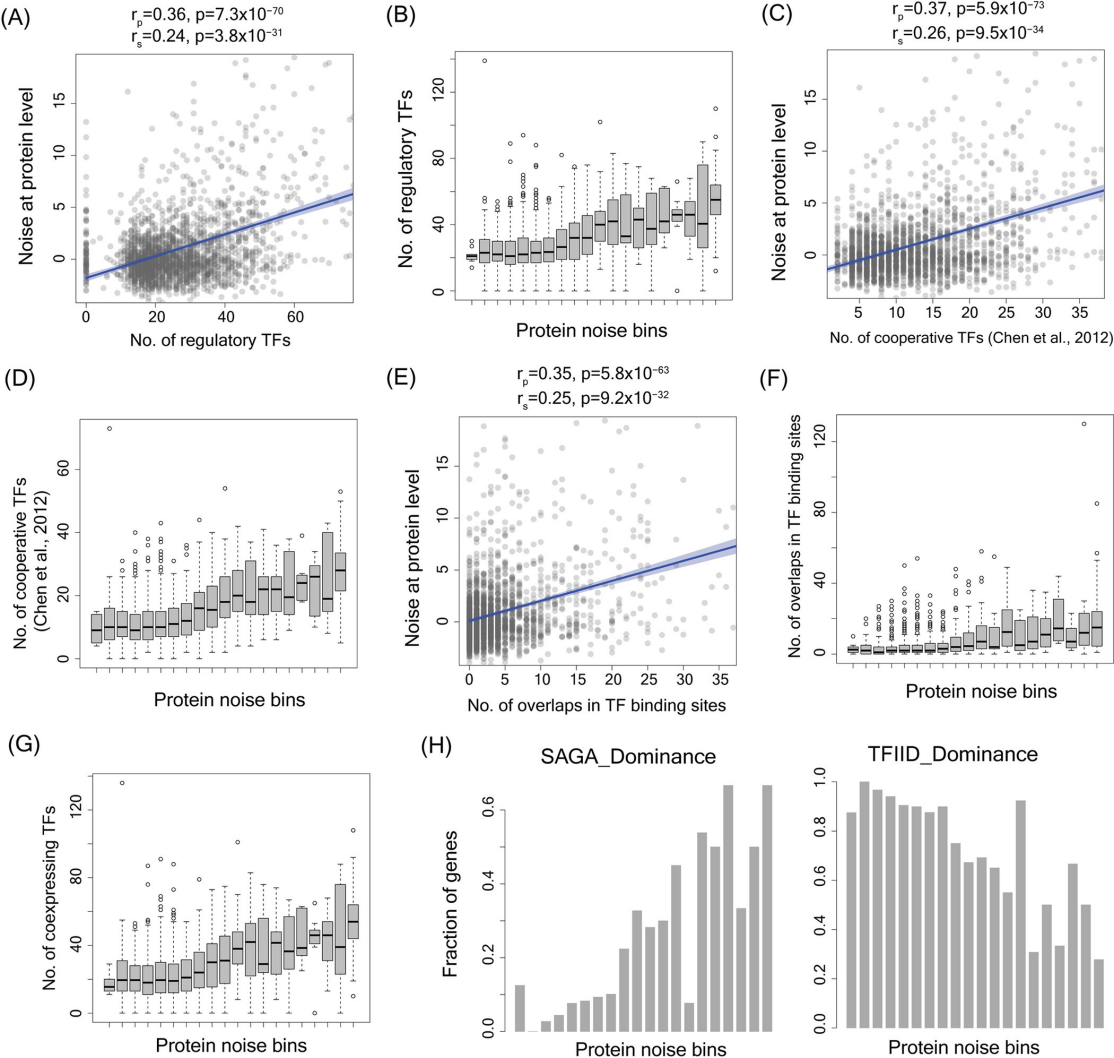
Genes with high expression noise were regulated by a higher number of TFs, had a higher number of cooperatively binding TFs, and showed more overlaps in TF binding sites compared to low-noise genes. **(A)** Correlation between noise at protein level and the number of regulatory TFs. **(B)** Number of regulatory TFs of genes across 20 protein noise bins. **(C)** Correlation between noise at protein level and the number of cooperative TFs [70] **(D)** Number of cooperative TFs of genes across protein noise bins. **(E)** Correlation between noise at protein level and the number of overlaps in TF binding sites. **(F)** Number of overlaps between TF binding sites for genes across protein noise bins. **(G)** Number of co-expressing regulatory TFs across protein noise bins. **(H)** Fraction of genes showing SAGA and TFIID dominance across protein noise bins.

Our model revealed a significant correlation between the number of regulating TFs of a gene and noise, at both mRNA and protein levels (for protein noise, Pearson’s correlation r_p_ = 0.36, p = 7.3×10^−70^ and Spearman’s correlation r_s_ = 0.24, p = 3.8×10^−31^; Fig 5A; for mRNA noise r_p_ = 0.26, p = 5.7×10^−85^; r_s_ = 0.19, p = 7.0×10^−47^; S6A Fig). We further classified genes into 20 equally spaced noise bins (barring the first and the last bins) sorted according to their noise values. The first bin had an open-ended lower limit for noise values to include genes showing very low noise levels. The last bin had an open-ended upper limit for noise values so as to include genes showing very high noise levels. This helped us avoid having bins with a very low number of genes. We then looked at the distribution of the number of regulatory TFs of genes in these bins (Figs 5B and S6B). The genes in the highest noise bins on average had >75% more regulatory TFs compared to the genes in the lowest noise bins (Figs 5B and S6B).

This raised a key question—how could an increase in the number of regulatory TFs lead to increased expression noise. Interestingly, genes regulated by a higher number of TFs showed a concomitant increase in the number of TFs exhibiting cooperative binding [69,70]. Expectedly, noise was significantly correlated with the number of cooperatively binding TFs for both mRNA and protein noise (Figs 5C and S6C. The genes in the highest noise bins on average had more than 66% cooperative TFs than the genes in the lowest noise bins (Figs 5D and S6D).

Further, an increase in the number of regulatory TFs and a corresponding increase in their binding sites resulted in a substantial increase in overlap of TF binding sites in the promoter region. This was reflected in the significant correlation between noise at mRNA and protein level with the number of TF binding site overlaps (Figs 5E and S6E). The median number of overlaps increased by more than 4-fold for genes in the highest noise bins compared to the genes in the lowest noise bins (Figs 5F and S6F). Cooperation and competition among TFs can occur only when the TFs are expressed at the same time inside a cell. Interestingly, genes in the highest noise bins on average had >90% increase in the number of co-expressing TFs than the genes in the lowest noise bins (Figs 5G and S6G). Further, genes in the highest noise bins had more than four times the fraction of SAGA dependent genes and had approximately three times lower number of TFIID dependent genes compared to the lowest noise bins, considering noise at both mRNA and protein levels (Figs 5H and S6H).

### Cooperative and Competitive TF binding could generate high expression noise

To better understand how an increase in the number of regulatory TFs can lead to higher expression noise, we built a mathematical model of gene regulation and performed stochastic simulations in a population of cells. Specifically, we asked whether a simple increase in the number of regulatory TFs could explain the higher expression noise and whether cooperative and competitive TF binding had any role to play in generating higher expression noise.

**Fig 6:**
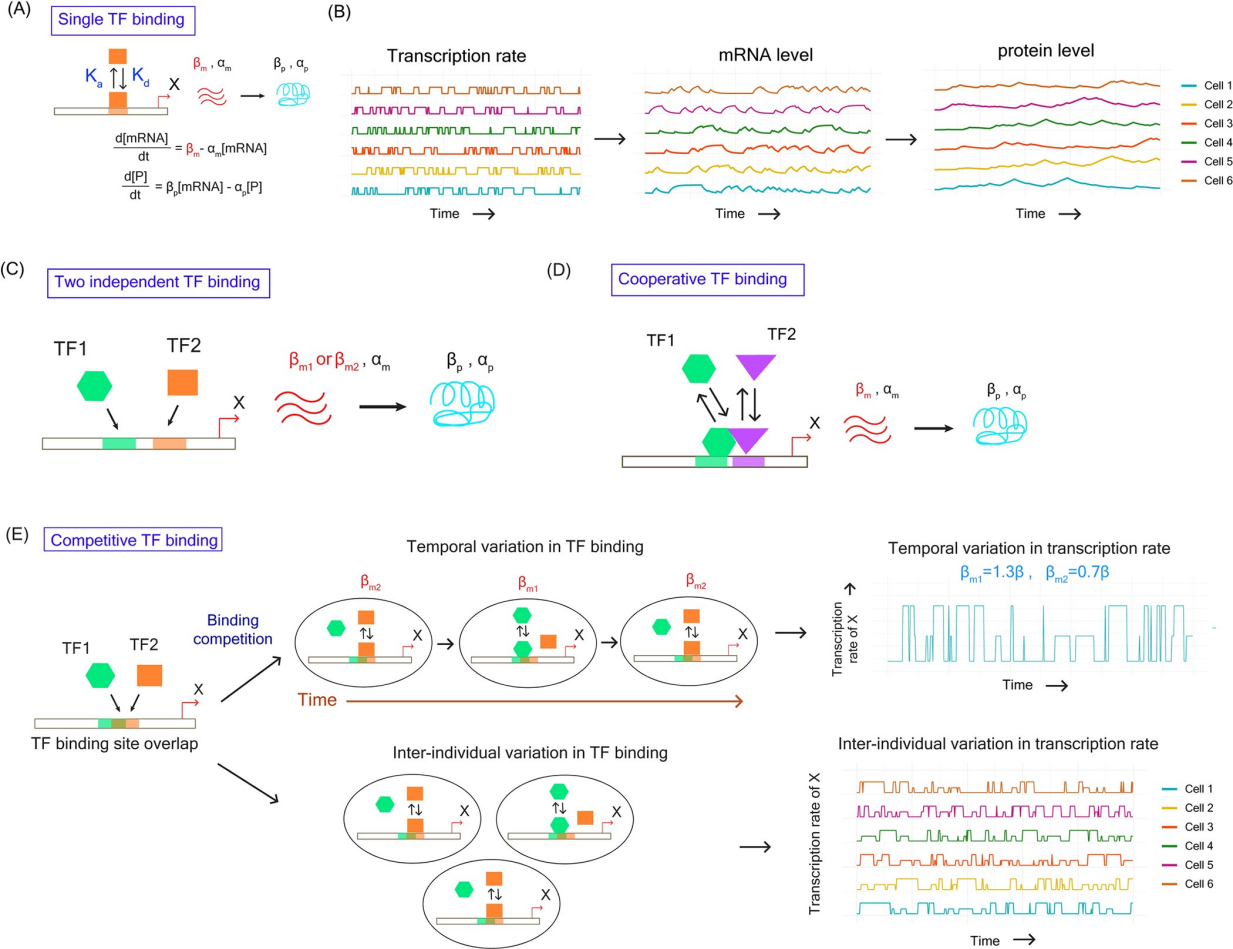
**Mathematical modeling and stochastic simulations of TF binding and impact on gene expression noise (A)** Mathematical representation of the model describing regulation by single TF **(B)** Schematic diagram showing the variation in transcription rate, mRNA levels and protein levels among individual cells obtained from mathematical modeling and stochastic simulation **(C)** Schematic diagram showing gene regulation by two TFs binding independently to the promoter. **(D)** Schematic diagram showing cooperative binding of two TFs to the promoter of a gene and induction of transcription. **(E)** Overlap between TF binding sites lead to binding competition between TFs. This could give rise to temporal variation in TF binding in the same promoter region within a cell. In addition, asynchrony in TF binding among individual cells could give rise to inter-individual variation in TF binding and transcription rate.

We first studied regulation of a gene by a single TF (Fig 6A). TF binding is a dynamic process consisting of rapid binding and unbinding steps [71,72]. Thus, we used a two-state model of gene expression where a gene could exist in on- and off- states with specific rates of transition between these two states. The binding of a TF resulted in transition to on-state that led to production of mRNA and proteins. We quantified variations in the gene expression levels over time by stochastic simulations using Gillespie’s algorithm (Fig 6B). We modeled the dynamics of gene expression in 10000 cells and quantified mean expression level and noise.

In the next step, we tested whether a simple increase in the number of TFs could impact expression noise. To do so, we modeled regulation of a gene by two TFs binding independently to the promoter region (without any cooperation or competition) (Fig 6C). Here we assumed that binding of any one of the TFs to the promoter led to the on state and resulted in production of mRNA and protein. When both the TFs were bound to the promoter, the transcription rate increased and was equal to the sum of the transcription rates for the individual TFs.

In cooperative binding of two TFs, we modeled the transcription rate by Hill function and assumed that the transcription as an all-or-none process regardless of the value of Hill coefficient. This meant that in cooperative binding of two TFs, substantial transcription occurred only when both TFs were simultaneously bound to the promoter region (Fig 6D). This process could alter the frequency of transcriptional bursts thereby affecting the overall mRNA and protein expression (S7 Fig). However, cooperative TF binding can prolong the duration of the on-state and can prevent transition to off-state [73]. We modeled this through a reduction in the rate of transition to the off-state. This allowed us to perform all comparisons of expression noise at similar mean expression levels (S7 Fig).

Competition among two TFs for binding to the overlapping sites in the promoter region could generate noise in two possible ways. First, competition between TFs could lead to a scenario where a gene would be regulated by different subsets of TFs at different points of time, thus generating temporal variation (Fig 6E). In presence of TFs that differ in their strengths of regulation, this could lead to temporal variation in transcription rate within a cell. Secondly, asynchronous temporal variation in TF binding among individual cells in a population could generate inter-individual variation in expression (Fig 6E).

**Fig 7:**
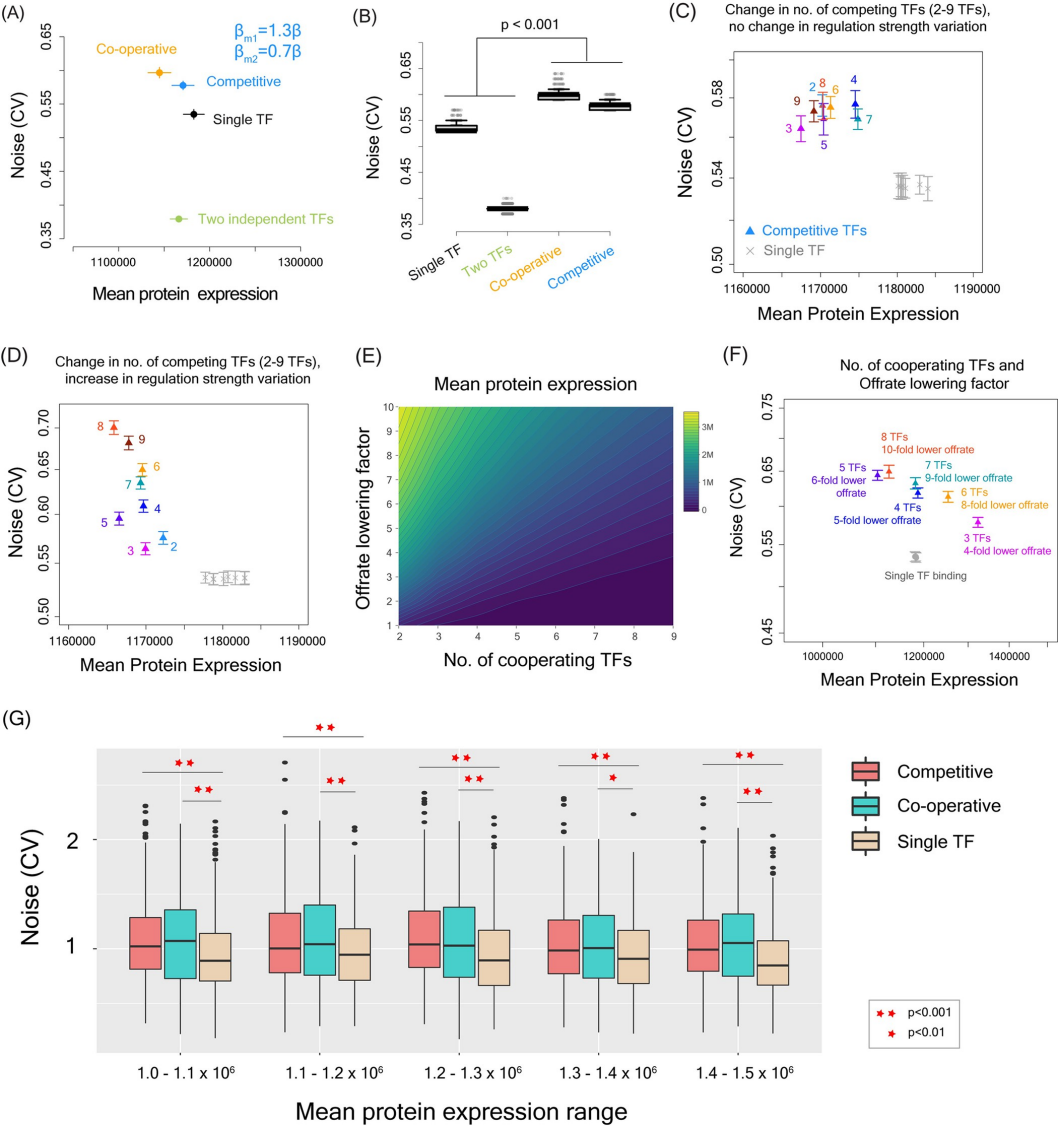
Mean expression and noise in case of gene regulation by a single TF, two independent TFs, two cooperative TFs and two competitive TFs. **(A)** Mean expression and noise values obtained from modeling and simulations of gene regulation by single TF (black), two TFs binding independently (green), two TFs binding cooperatively (orange) and two TFs binding competitively (blue). **(B)** Noise distribution in cases of gene regulation by a single TF and by two TFs binding independently, cooperatively and competitively. Noise was calculated across multiple time-points in 10,000 simulated cells. **(C-D)** Changes in expression noise with an increase in the number of competitive TFs with and without changes in the variation in regulation strength. **(E)** Increase in the number of cooperating TFs can drive mean protein expression down without any change in the on and off-rates. However, the expression remains the same if binding of more cooperative TFs can increase the time that a gene remains in the on state by lowering the off-rate. **(F)** Gene expression noise in case of 3–9 cooperative TFs at the similar mean protein expression level. The off-rate parameter was adjusted to achieve similar range of mean expression in all cases. **(G)** Boxplots showing noise values in single TF, competitive TF and cooperative TF binding from stochastic simulations with Markov-Chain Monte-Carlo sampling of parameters of the mathematical model. The target mean protein expressions were set between 1×10^6^ and 1.1×10^6^, between 1.1×10^6^ and 1.2×10^6^, between 1.2×10^6^ and 1.3×10^6^, between 1.3×10^6^ and 1.4×10^6^, between 1.4×10^6^ and 1.5×10^6^ molecules.

Interestingly, at similar mean protein expression levels, regulation by two independent TFs had lower noise than single TF regulation (Fig 7A and 7B), as the target gene was more frequently in the on-state by the action of one of the two TFs and therefore, had less temporal and inter-individual variation in the protein level. This demonstrated that a simple increase in the number of regulatory TFs could not explain the higher noise observed in genes with higher number of regulatory TFs. In comparison, both cooperative and competitive binding of TFs led to higher noise compared to regulation by a single TF or by two independent TFs (Fig 7A and 7B), suggesting that the dynamics of TF binding process in case of gene regulation by multiple TFs has an important role in generation of expression noise.

We further explored whether variations in the parameters of the model such as transcription and translation rates, degradation rates, number of cooperative and competitive TFs could influence our inference (S1 Text). We first performed a mathematically controlled comparison between single, competitive and cooperative TF binding where we kept all model parameters the same across these different scenarios and only varied the TF binding process. We did so to understand the contribution of the TF binding process on expression noise and to avoid confounding our results by variations in other parameters that could also influence expression noise. We performed stochastic simulations with choice of model parameters over a broad range of parameter values in a mathematically controlled manner across single TF, competitive TF and cooperative TF binding scenarios. Over these broad range of parameter values, competitive and cooperative TF binding showed higher noise compared to single TF regulation (S8 and S9 Figs).

We extended our analysis to quantify noise in cases of cooperation or competition between more than two TFs as many transcription initiation complexes can contain multiple TFs. Competitive TF binding showed higher noise compared to single TF regulation regardless of the number of TFs and regardless of change in regulation strength among TFs (Fig 7C and 7D). For cooperative TF binding, increase in the number of TFs resulted in a reduction in burst frequency and a reduction in the mean protein level (S7 Fig). However, an increase in the number of cooperating TFs could proportionally increase the time being spent in the on- state which we modeled through a reduction in off-rate so as to maintain similar mean protein level even with an increase in the number of cooperating TFs (Fig 7E). In all these scenarios, noise was higher in case of regulation by cooperative TFs compared to a single TF regulation (Fig 7F). This suggested that inferences drawn from our models hold regardless of the number of TFs involved in cooperative and competitive binding.

Overlaps in binding sites of different TFs can also lead to degeneracy of TF binding sequences to accommodate diverse consensus binding motifs of different TFs. Such degeneracy can change binding affinity of TFs to the DNA and can lead to noisy transcription. However, we did not see any difference in binding site degeneracy among the promoter regions of genes with high and low expression noise (S10 Fig).

To further test whether these results hold over any combination of parameter values with the only condition of the mean protein expression being the same across single, competitive and cooperative TF binding, we performed a Markov-Chain Monte-Carlo (MCMC) sampling of parameter space. Briefly, we did model initialization with a set of random parameter values for all parameters of the model, followed by parameter optimization so as to reach a target mean expression level. At each step, we performed stochastic simulations over 1000 cells in each of single, competitive and cooperative TF binding and calculated mean expression. Once the simulation reached the target mean expression level, we also calculated noise values. We chose five different target mean expression levels for comparison (Fig 7G) that were in a reasonable range of burst size and burst frequency. This avoided comparing noise in expression states where a gene was always on or always off. Over all these mean expression levels, competitive and cooperative TF binding showed significantly higher expression noise value compared to single TF binding (Fig 7G).

## Discussion

In summary, through an integrated statistical analysis we have shown that the transcription factor binding process is the most important contributor to gene expression noise. Although many earlier studies have investigated the molecular origins of expression noise, most of them have focused on the role of the TATA box sequence, promoter nucleosome occupancy patterns, and histone modifications. Although earlier work and our analysis found significant association between presence of the TATA box sequence and expression noise in yeast, such association was not observed by Wu *et al*. [34] in human embryonic stem cells. Here we show that, despite the strong association, presence/absence of TATA box sequence and promoter nucleosome occupancy are not good predictors of expression noise in yeast. Instead, our work uncovers an important role for TFs in noise regulation. We show that noisy genes tend to be controlled by a larger number of TFs. These include a substantial fraction of TFs that bind cooperatively to the promoter region. In addition, an increase in the number of regulating TFs can cause an increase in overlap among the TF binding sites which can lead to competition between TFs for binding to the same promoter region. This can give rise to temporal as well as inter-individual variation in TF binding, thereby increasing noise.

An earlier work has shown that an increase in the number of transcription factor sites can increase gene expression noise [32]. This study found that the number of TF binding sites and their spacing could influence noise, as could the insertion of a nucleosome disfavouring element. They also observed that the larger and denser clusters of TF binding sites led to higher noise, thus suggesting that the competition between TFs could possibly result in higher expression noise. However, this study focused only on TATA-containing promoters and binding sites of two activators, GCN4 and LEU3. Thus, the conclusions drawn from their work might not be applicable to non-TATA promoters or to the wide variety of transcription factors present in yeast. Nevertheless, their work provided some experimental evidence of the influence of competitive TF binding on expression noise. Further, their experimental design could be a template for further experiments to understand how cooperative TF binding can lead to higher expression noise which has not been explored so far.

Another study found that competition between interacting partners of the TATA binding protein influences noise [38] but this was limited to the TATA binding protein (TBP). In contrast, our analysis considered all possible promoter sequences and transcription factors and demonstrated that transcription factor binding process is the key driver of expression noise. Thus, we describe a general molecular mechanism of noise generation that is not dependent on any specific TF or any specific promoter sequence.

An earlier study by Faure *et al*. [35] analyzed expression noise in mouse embryonic stem cells and looked into the role of several molecular features in noise regulation. They analyzed the role of histone modification patterns, super-enhancer regions along with promoter sequence features such as transcription initiation sites and presence of the TATA box motif. Through quantification of effect sizes, they observed association of some of these features to expression noise. In addition, they assessed the relative importance of these molecular features, individually as well as in combinations, in classifying genes into high- or low-noise categories. However, the authors did not report the fraction of variation explained or the predicted R^2^ values. In contrast, our integrated statistical model specifically reported the predictive capabilities of the molecular features, individually as well as in combination. This enabled us to quantify how well presence or absence of a molecular feature individually or in combination with other features in a gene or its promoter could predict expression noise of that gene.

An interesting observation from our integrated models was that the performance of the model for prediction of mRNA noise was similar to the performance of the model for prediction of protein noise. This is despite the fact that measuring mRNA expression in single cells is technically more difficult and less precise as compared to measuring protein expression in single cells, as the quantification of mRNA levels in single cells suffers from poor capture efficiency and sampling effects. We believe that there are two possible reasons which can explain the comparable performance of the mRNA noise prediction model and the protein noise prediction model. First, we had substantially more data available in the mRNA noise dataset (~5500 genes) compared to the protein dataset (~2800 genes). This means that the predictive model for mRNA noise had a substantially bigger training dataset which helped in building a model with performance similar to the protein noise prediction model. Second, our features, to a great extent, focused on mRNA synthesis and decay rates, mRNA stability, which directly impact expression noise at the mRNA level but only indirectly influence protein noise.

Although our integrated model could predict a substantial fraction of noise variation, there was, however, still a large fraction of noise that could not be explained by our model. This can be due to several reasons. Firstly, it is possible that several other molecular features which can regulate noise have not been considered in our model. Some of these molecular features may still be unknown. Second, there is inherent randomness in molecular processes occurring inside a cell and expression noise can also vary with time. Thus, our calculation of noise at a single time point data may also impact predictive power. Third, the experimental data on molecular features considered in this study have been obtained from different research groups and in different growth conditions. This can impact the predictive ability of our model. Fourth, we understand that some of the features such as the nucleosome occupancy levels and histone modification patterns are dynamic in nature and can change with time. As we modeled these features using datasets obtained at a single time-point, we might have completely missed the contribution by dynamic nature of these features in noise regulation. Further, growth conditions, growth rate of cells and cell cycle have all been observed to influence gene expression noise [74–76]. Thus, combining data on molecular features across many datasets without consideration for these variables can potentially affect predictive power. Finally, on the modeling side, we used a linear regression model for our analysis which is able to capture linear trends in the data but might miss non-linear associations present in the data. This might affect model performance. However, to counter this drawback we also used a random forest model which is able to capture non-linear trends in the dataset.

In summary, our findings provide a step forward for prediction of expression noise. Recent explosion in genomic data has led to genome-wide characterization of TF binding sites across a diverse range of organisms. In addition, with increasing availability of genome-wide nucleosome occupancy maps, histone modification patterns and three-dimensional genome configuration data, our study provides a framework for building integrated models of gene expression noise in other organisms in future. Stochastic variations in molecular processes are ubiquitous in cells across biological systems and have major implications for human diseases. Thus, an enhanced ability to predict variations in biological processes will be extremely useful in quantifying the extent of heterogeneity in cellular traits and phenotypes.

## Methods

### Calculation of expression noise for individual genes

Noise values for individual genes in yeast at the protein level were obtained from Newman *et al*. [19] and the DM values in the YPD medium were used for expression noise analysis. The DM values in the YPD medium were highly correlated with DM values obtained in the SD medium. The noise values of all genes at the mRNA level were calculated from the single-cell RNA-seq data provided by Nadal-Ribelles *et al*.[49] as follows. Briefly, for each gene the coefficient of variation (CV) was calculated from its mean expression and standard deviation value. Different polynomial fits were made to the CV vs log-transformed mean expression value and the best fit was chosen. A polynomial of order 5 was found to give the best fit. The mean adjusted noise value for a gene was obtained by calculating the vertical distance between the CV value and the best fitted curve. To estimate the impact of outliers on fitting, 95% confidence intervals for the fits were also estimated and were plotted along with the fitted line.

### Building an integrated model of expression noise in yeast

The integrated model of noise was generated by considering a total of 329 molecular features. These features could potentially impact gene expression and therefore, could also influence expression noise. These features included sequence features, epigenetic modifications, transcription factor binding, mRNA and protein properties. Data on all features were obtained from published work.

### Promoter sequence features

As genes involved in stress response have earlier been shown to be noisy, we considered presence of the STRE elements in promoters as one of the first features in our model. The STRE elements are required for binding of stress responsive TFs *MSN2* and *MSN4* to the promoters of the stress response genes in yeast. Data on presence of the STRE elements were taken from Moskvina *et al*. [77]. Presence of the TATA box sequence in the promoter region of a gene has been strongly associated with higher expression noise. Therefore, presence/absence of the TATA box sequence was a molecular feature in our model and the data on promoters with TATA box sequence was obtained from [67].

### Gene sequence features

Transcription initiation regulates the overall expression of a gene and the location of the transcription start site (TSS) is important in this regard. In addition, if a gene has multiple TSS sites, this can be a potential source of expression variation between individual cells of a population. TSS data for all yeast genes were obtained from [78]. Closest TSS site for each gene was obtained, and a spread of potential TSS sites for all genes was calculated.

tRNA adaptation index (tAI) measures translational efficiency and is dependent on availability of tRNA molecules in an organism. tAI can thus be an indicator of expression levels of genes. tRNA adaptation index for all genes was calculated following the method of [61]. The tAI values for first 5, 10, 15, 20, 25, 30, 40 and 50 codons were calculated along with the tAI value for the whole gene, as the first few codons can have major influence on gene expression level and hence on noise.

### Features associated with nucleosome occupancy and histone modifications

Nucleosome occupancy patterns in promoter regions can influence TF binding and thus, can impact the transcription process. Genome-wide absolute nucleosome occupancy level for yeast was obtained from Oberbeckmann *et al*. [66]. The number of nucleosome-occupied sites and the absolute nucleosome occupancy level per nucleosome-occupied site for promoter regions and gene bodies were calculated. The region from 1000bp upstream to 10bp downstream of the start codon of a gene was considered to be the promoter region of the gene. Average nucleosome occupancy level per occupied site calculated between -1000bp to -900bp region of the start codon was shown as the nucleosome occupancy at -1000bp. Similarly, average nucleosome occupancy level per site was calculated for -900 to -800bp, -800 to -700bp, -700 to -600bp, -600 to -500bp, -500 to -400bp, -400 to -300bp, -300 to -200bp, -200 to -150bp, -150 to -100bp, -100 to -50bp and -50bp to +10bp regions.

Histone modification patterns have been associated with expression noise in earlier studies. Genome-wide histone modification data for yeast were obtained from Pokholok *et al*. [58] and all different types of modifications were mapped to gene bodies, promoter regions, and transcription factor binding sites. Histone binding dynamics can also influence gene expression and therefore, expression noise. Thus, the histone binding dynamics data obtained from Dion *et al*. [59] were used as molecular features in our model and all different measures described in their paper were considered.

### mRNA and protein features

The synthesis rates and decay rates of mRNA and proteins are important determinants of expression levels of genes. The mRNA synthesis rates and decay rates were obtained from Sun *et al*. [79]. Data on mRNA secondary structures in yeast were obtained from Kertesz *et al*. [62]. The mRNA half-life data and the protein half-life data were obtained from Geisberg *et al*. [63] and Belle *et al*. [64] respectively.

Post-translational modifications of proteins can impact expression levels of genes and can be a source of variability in gene expression among individual cells of a population. Data on post-translational modifications in yeast were obtained from YAAM database [65] and the numbers of different types of modifications for each protein were calculated.

### Analysis of transcription factor binding

The list of transcription factors for all yeast genes was obtained from Yeastract database (http://www.yeastract.com/) [80]. For a gene, only those TFs for which experimental evidence for DNA binding had been obtained or the knockout of TF had been experimentally shown to impact expression of the gene were considered. In addition, the binding sites of all TFs to promoter regions of the target genes were searched and mapped using the consensus motif sequences for TFs obtained from YeTFaSCo database [81]. All position weighted matrices for all motifs were obtained and all possible combinations of bases were considered. Positions of all such motif sequences of all regulatory TFs of a gene were identified in the promoter region (ranging from -1000bp to +10bp of the start codon) after allowing for maximum two mutations in the consensus sequence.

### Mean expression and noise levels of TFs

Several features related to transcription factors were included in our model. As TFs could have different expression noise distributions compared to non-TF genes, whether a gene was a TF could be an important determinant of noise. The number of regulatory TFs for genes was included as a feature. If the regulatory TFs show noisy expression, this could generate large inter-individual variation in expression of target genes. Therefore, the median expression level, noise level, positive and negative noise levels (DM values) of regulatory TFs were considered as features in the model. In addition, the percentage of TFs showing low and high noise values, their minimum and maximum noise values were considered. The expression noise could be generated by activators or repressors or by simultaneous regulation of both. Therefore, the numbers and percentages of activators and repressors were considered. In addition, the ratio of the number of activators to repressors for each gene and the noise levels of activators and repressors were considered as features.

### TF regulation strength and TF co-expression

Strength of regulation by TFs strongly impacts expression levels of target genes. Thus, the mean and standard deviation values of regulation strength of activators and repressors were considered as features. Co-expressing TFs can influence the expression level of a gene through synergistic or antagonistic effects. Thus, the number and percentage of TFs showing positive as well as negative expression correlations were included as features. Co-expressing TFs when competing for binding to the overlapping sites in the promoter sequence can be a source of inter-individual variation in expression level. Therefore, the percentage of co-expressing TFs binding to overlapping sites in the promoter sequence was considered.

Mutations in the TF binding motifs can impact strength of gene regulation and thus, can affect gene expression level. The number of TF binding sites at different distances upstream of the start codon can exert different levels of regulation strength. This can influence expression noise. Therefore, the number of TF binding sites up to 100bp upstream region of the start codon, between 100 to 200bp, between 200 to 300bp, between 300 to 400bp, between 400 to 500bp, between 500 to 600bp, between 600 to 700bp, between 700 to 800bp, between 800 to 900bp and between 900 to 1000bp upstream regions of genes were considered as individual features in our model. In addition, the mean expression and expression noise of the TFs binding at different distances upstream of the start codon were also considered. Further, the levels of nucleosome occupancy as well as histone modification patterns in the TF binding sites were considered as features in our model. Moreover, the percentage of nucleosome occupancy and histone modification levels in the TF binding sites as compared to the whole promoter region were included as features.

### Competitive and Cooperative TF binding

Overlaps in TF binding sites in the promoter sequence of a gene can lead to competition between TFs for binding to the same promoter region. This can, in turn, generate inter-individual variation in gene expression. Therefore, the number of overlaps between TF binding sites, the ratio of the number of overlapping sites to the total number of TF binding sites, the number of overlaps at different distances upstream of the start codon and the average length of these overlaps were included as features. In addition, percentage of overlapping sites shared by two activators, shared by two repressors, and shared by an activator and a repressor were considered. Furthermore, the average strength of regulation as well as the differences in strength of regulation for all the above cases were included as features in our model. Cooperative binding of TFs can impact the rate of transcription and can thus determine gene expression level. Therefore, the number and the percentage of cooperatively binding TFs were included from the list of cooperatively binding TFs in yeast from Yang *et al*. [69] and Chen *et al*. [70].

### Broad-acting TFs and 3D genome configuration

The transcriptional activators SAGA and TFIID are important components of RNA polymerase complex and influence transcription initiation. The classification for SAGA or TFIID dependence of genes for their expression was obtained from Huisinga and Pugh [50]. In addition, co-activator redundant or TFIID dependent classification of genes was used from Donczew *et al*. [51]. Along similar lines, broadly acting TFs can impact expression levels of genes. The binding activities of several broadly acting TFs such as TBP, ABF1 and RAP1 were obtained from van Werven *et al*. [52], Lickwar *et al*. [53] and de Jonge *et al*. [54], respectively. Chromatin remodelers influence binding of TFs to DNA and can thus influence gene expression. Binding patterns of chromatin remodelers were obtained from Yen *et al*. [55], Zentner and Henikoff [56], and Ramachandran *et al*. [57].

The three-dimensional (3D) configuration of the genome can influence DNA accessibility and long-range interactions between regulatory elements. Therefore, the 3D genome configuration was considered as a feature in our model. Data on three-dimensional model of yeast genome were obtained from Duan *et al*. [60]. Number of intra- and Inter- chromosomal contacts for all genes and promoter regions were quantified.

### Regression analysis

The integrated dataset was first scaled using z-score standardization, and the fraction of variation explained and the predictive capability of each molecular feature were quantified by linear regression. To perform linear regression, the function ‘lm’ in R was used. For quantifying predictive ability of features individually, expression noise was modeled as: $Noise=\beta_{0}+\beta_{1}\times feature+\varepsilon$, where ‘ε’ represents error.

For estimating the predictive power of a set of features on noise, variable selection using Ridge and Lasso regression were performed to minimize the problems of multi-collinearity and overfitting. Ridge regression was performed with the R package ‘ridge’ [82], appropriate number of principal components was chosen and features showing significant effect on noise were identified. Lasso regression was performed using the R package ‘glmnet’ [83]. The best lambda value was obtained by a 10-fold cross-validation and the lambda for which the cross-validation error was minimum was chosen for subsequent steps. For the preferred lambda value, features whose model coefficients showed non-zero values were considered as features influencing noise. The most important features were further chosen through a stepwise addition and removal process using stepwise regression where Akaike Information Criterion (AIC) of the fitted models were minimized. This was done using the R package ‘olsrr’ (https://github.com/rsquaredacademy/olsrr). The features in the model with lowest AIC values were selected for further analysis.

In the next step, linear regression with the selected features was performed on the training set to obtain the fraction of variation explained. Specifically, expression noise was modeled as:

$$Noise=\beta_{0}+\beta_{1}\times feature1+\beta_{2}\times feature2+\beta_{3}\times feature3+\ldots +\beta_{n}\times feature\;n+\varepsilon$$

The coefficients (β_i_ values) of the model were estimated from the training data. The linear regression model obtained was then applied on the test data to obtain predicted noise values along with predicted R^2^. The process of dividing dataset, training and prediction was repeated 1000 times to obtain mean and standard deviation values for fraction of variation explained and predicted R^2^ values. Further, random forest models were also built using the selected features using the R package ‘randomForestSRC’ both with and without missing value imputations. These also resulted in fraction of variation explained and predicted R^2^ values.

In addition to the original dataset, two filtered datasets were created with reduced number of variables–one filtered on correlation and another filtered on impact. The first filtered dataset was created by removing features that did not show significant correlation (p<0.05) with noise. To create the second filtered set, first, the impact of all individual features on noise were obtained by linear regression as described above. Only the features that showed significant impact (explained at least 0.05 fraction of the noise variation or had predicted R^2^ of at least 0.05) were retained in the filtered set. Linear regression was performed on these datasets as described above to obtain fraction of variation explained and R^2^ values for prediction. The analysis showing best results for fraction of variation explained and predicted R^2^ was reported.

### Gene-transcription factor (TF) and TF-TF expression correlation analysis

Gene expression data measured through RNA sequencing from Dhar *et al*. [84] (NCBI GEO dataset id 104343) was used to calculate all pairwise gene-TF and all pairwise TF-TF (of a gene) expression correlations. Significant positive correlation (p<0.05) between a gene and a regulatory TF indicated that the TF acted as an activator for the gene since the expression of the gene increased with increase in expression of the TF. Similarly, significant negative correlation between a gene and its regulatory TF indicated that the TF acted as a repressor for the gene. The value of correlation coefficient between a gene and a TF (if significant) was taken as the response correlation and the slope of the line was considered as the strength of regulation of the TF. In addition, pairwise expression correlations between all TFs of a gene were calculated. If a TF showed significant positive correlation with at least three other TFs, the TF was considered to be a positively correlated (co-expressing) TF. Similarly, if a TF showed significant negative correlation with at least three other TFs, the TF was considered to be a negatively correlated TF.

### Modeling and stochastic simulation of TF-DNA binding process

The dynamics of TF binding to DNA was studied using a two-state model with consideration for rapid binding and unbinding of TF to DNA. The binding-unbinding of a TF to DNA was considered to be a Poisson process and thus, the time intervals between two successive bindings (or two successive unbindings) were exponentially distributed. The time intervals between successive events (on or off switching) were sampled from exponential distributions with rate parameters denoted as λ_on_ and λ_off_ respectively. The dynamics of cooperative binding and competitive binding of TFs was compared to the dynamics of regulation by a single TF and two independent TFs. For modeling binding of two TFs, on- and off-time intervals were sampled from Poisson distributions individually for each of the TFs with the same rate parameters. For cooperative binding, only when both the TFs were bound to the promoter, the gene switched to the on state and led to production to mRNA and protein molecules at the same rates as the single TF binding. This resulted in lowering of burst frequency in case of cooperative TF binding which eventually reduced the mean protein level. To address this issue, λ_off_ was gradually reduced to achieve similar mean expression level as in single TF regulation. Reduction of λ_off_ values prolong the on-state in cooperative TF binding [73]. Competitive TF binding was modeled in the same way as modeling single TF binding but TF that bound to the promoter at every on-state transition was randomly chosen. The rate of production of mRNA was influenced by the regulation strength of the TF that bound to the promoter.

Binding of a TF led to switching to on state which resulted in production of mRNA at a rate β_m_ and translation of these mRNA molecules to proteins at a rate β_p._ These mRNA and protein molecules were considered to undergo removal resulting from dilution due to cell growth and degradation at the rates of α_m_ and α_p_ respectively.

The dynamics of transcription and translation were modeled using the following equations.

$$Changes\;in\;mRNA\;conc.\;over\;time:\;\frac{d[mRNA]}{dt}=\beta_{m}–\alpha_{m}\times [mRNA]$$

where β_m_ denoted the transcription rate per unit time (or burst size) and α_m_ denoted the removal rate of mRNA due to degradation and dilution.

$$Similarly,\;Changes\;in\;protein\;conc.\;over\;time:\;\frac{d[P]}{dt}=\beta_{p}\times [mRNA]–\alpha_{p}[P]$$

where β_p_ denoted the protein production rate from mRNA and α_p_ denoted the protein removal rate.

All rate parameters for single TF, two independent TF, cooperative TF and competitive TF binding were chosen in such a way that the comparisons were mathematically equivalent. As the concentrations of TFs can impact the chances of binding, the concentration of TF in single TF binding scenario was considered to be the same as the concentration of each of the cooperatively binding TFs. Further, the concentration of the TF in single TF binding scenario was considered to be equal to the sum of the concentrations of two TFs in case of competitive binding scenario. The transcription rates in the cases of regulation by single TF and by cooperative TFs were exactly the same. The transcription rates in case of regulation by two independent TFs were chosen in such a way that the average transcription rate was equal to the transcription rate in regulation by a single TF. The transcription rates of the TFs in case of competitive TFs were chosen in such a way that the average transcription rate of the two TFs was the same as the transcription rate in single TF regulation. Therefore, the parameters β_p_, α_m_, α_p_ were considered to be the same across all cases of TF binding. In case of cooperative TF binding, β_m,coop_ was assumed to be the same as the β_m,single._ In case of competitive TF binding, the production rates varied between two TFs, with β_m1_ = 1.3×β_m,single_ and β_m2_ = 0.7×β_m,single_.

Stochastic simulations were performed using Gillespie’s algorithm [85] to decipher the dynamics of TF-DNA binding in all scenarios and to investigate the impact of cooperative and competitive TF binding on noise. The behavior of the system was tracked at small discrete time intervals Δt from the initial time point t. These resulted in observations at ‘n+1’ time points t, t+ Δt, t+2×Δt, …, t+n×Δt. Any event of binding or unbinding occurring within a time interval was noted and resulted in changes in transcription rate which eventually led to a change in protein concentration. Binding of TFs led to transcription and increase in mRNA and protein concentration according to the above equations. As the time interval Δt was considered to be small, the equations modeling the behavior of the systems was simplified as

$${\left[mRNA\right]}_{t+\Delta t}={\left[mRNA\right]}_{t}+(\beta_{m}–\alpha_{m}\times {\left[mRNA\right]}_{t})\times \Delta t$$

and

$${\left[P\right]}_{t+\Delta t}={\left[P\right]}_{t}+(\beta_{p}\times {\left[mRNA\right]}_{t}–\alpha_{p}\times {\left[P\right]}_{t})\times \Delta t$$

β_m_, α_m_, β_p_ and α_p_ were expressed in appropriate units for further simplification of these equations. The dynamics of transcription, variation in the mRNA concentration and variation in protein concentration with time were modeled across 10,000 cells. Noise was expressed as coefficient of variation (CV) from the calculation of mean and standard deviation in the protein level across these 10,000 cells and across all individual time points.

Mean expression level and noise values were calculated for a wide range of parameter values for all the parameters λ_on_, λ_off_, β_m_, β_p_, α_m_, and α_p_ to ensure that the results obtained were not biased by the choice of specific parameter values. All noise comparisons were made at similar mean expression levels to eliminate any bias in the noise values due to variations in mean expression levels. This was done following two approaches. In the first approach, only one of the parameters was varied while keeping others constant across the scenarios of single, competitive, and cooperative TF binding. This allowed us to do mathematically controlled comparison across single, competitive and cooperative TF binding with difference existing only in the TF binding process. In the second approach, a Markov-Chain Monte-Carlo (MCMC) sampling of the model parameters was performed to explore the high-dimensional parameter space while keeping mean expression level similar across single, competitive and cooperative TF binding.

### MCMC sampling of parameter space

Since the model had multiple parameters and each with a range of possible values, the combination of possible parameter values was large and the parameter space was high-dimensional. Thus, it was not possible to calculate expression noise values for all possible parameter combinations. Therefore, an MCMC sampling of the parameter space was performed with the target of achieving the same mean expression level for single, competitive and cooperative TF binding. To do so, the model was first initialized with a random set of parameter values so that the mean expression level was within the five times the target mean expression value. This was done to ensure that a convergence to the mean expression value could be reached within a reasonable number of iterations. For each of the model parameters, the minimum and the maximum values and the step size for change were defined.

In the next step, one of the parameters was randomly changed according to the pre-defined step size and the change in mean expression was quantified from the model. If the change in the parameter value took the mean expression of the model closer to the target value, the change in the parameter value was accepted and the next change was performed in the same parameter value in the same direction. If any change in a parameter value took the mean expression level away from the target value, the change in the parameter value was rejected, if the change took the mean expression value beyond two times the target mean expression range and a new parameter was randomly chosen for the next step. This process was repeated until convergence or up to a maximum of 50 iterations. At each step, the mean expression level and noise was calculated based on analysis in 1000 cells for each of single, competitive and cooperative TF binding. For each of single, competitive and cooperative TF binding, 10000 independent MCMC samplings were performed and the mean protein expression level and expression noise values were reported. The target mean protein expression levels were chosen to be between 1 × 10^**6**^ and 1.1 × 10^**6**^ molecules, between 1.1 × 10^**6**^ and 1.2 × 10^**6**^ molecules, between 1.2 × 10^**6**^ and 1.3 × 10^**6**^ molecules, between 1.3 × 10^**6**^ and 1.4 × 10^**6**^ molecules and between 1.4 × 10^**6**^ and 1.5 × 10^**6**^ molecules. Only cases with the burst frequency between the values 0.2 and 0.8 were considered for our analysis, as otherwise a gene was in always off or always on mode.

### Modeling the impact of increase in number of TFs on expression noise

As competitive and cooperative TF binding can involve more than two TFs, the impact of an increase in the number of competitive or cooperative TFs on expression noise was quantified through our model. The number of TFs was varied from two to nine TFs. In case of competitive TF binding with multiple TFs, the rate of transitions to ‘on’ or ‘off’ states remained unaltered. However, there were more TFs available for binding to the same site in the promoter region and only one of the TFs could bind to the promoter site. The TF that could bind to the promoter site was randomly chosen. In case of cooperative TF binding with multiple TFs, the transcription was modeled as a Hill function and the transcription was assumed as an all-or-none process regardless of the value of Hill coefficient. Therefore, only binding of all TFs led to substantial transcription. This, however, led to substantial reduction in burst frequency and thereby reduced mean expression. To compensate for this, the rate of transition to ‘off’ state (λ_**off**_) was gradually reduced to achieve similar mean expression level as in single TF regulation. Reduction of λ_**off**_ values prolonged the on-state in cooperative TF binding [73] and thus, increased mean expression level.

## Supporting information

S1 Text Cooperative and Competitive TF binding caused higher noise across a wide range of model parameter values. (PDF)

S1 Fig **Distribution of expression noise of genes at the mRNA level (A) and at the protein level (B)**.
(TIF)

S2 Fig Presence of the TATA box sequence and the promoter nucleosome occupancy were associated with expression noise. **(A)** Difference in expression noise of genes with and without the TATA box sequence in the promoter, calculated at the mRNA as well as the protein level **(B)** Correlation between noise and average promoter nucleosome occupancy.
(TIF)

S3 Fig Distributions of feature values. Plots showing distributions of **(A)** number of regulatory TFs, **(B-C)** number of cooperative TFs [69,70], **(D)** number of TF binding sites, **(E)** number of TF binding site overlaps, **(F)** number of overlaps per TF binding site, **(G)** number of TFs showing positive expression correlation among themselves, **(H)** number of genes with SAGA dominance in the promoter, and **(I)** number of genes with TFIID dominance in the promoter.
(TIF)

S4 Fig Comparison of the fraction of variation explained and the predictive ability of the statistical models on datasets with and without duplicate genes. **(A)** Correlation between average fraction variation explained and the average rank for all features with and without the duplicate genes in the data. Average fraction variation explained and average rank for a feature were calculated by taking average of values in mRNA and protein noise data. **(B)** Correlation between average predicted R^2^ values and the corresponding average rank with and without the duplicate genes in the data.
(TIF)

S5 Fig Fraction of variation explained and predictive ability (given by predicted R^2^ value) in models with and without duplicate genes. Fraction of variation explained and predictive ability of features associated with TF binding activity, combination of TF binding activity with other features, combination of all features excluding PTMs and combination of all features including PTMs. The ‘+’ and ‘-’ signs denote the datasets used in analysis, with ‘+’ indicating the full dataset and the ‘-’ sign indicating the dataset after removal of duplicate genes.
(TIF)

S6 Fig Genes with high expression noise at mRNA level were regulated by a higher number of TFs, had higher number of cooperatively binding TFs, and showed more overlaps in TF binding sites compared to low-noise genes. **(A)** Correlation between noise at mRNA level and the number of regulatory TFs **(B)** Number of regulatory TFs of genes across different mRNA noise bins **(C)** Correlation between noise at mRNA level and the number of cooperative TFs [70] **(D)** Number of cooperative TFs of genes across mRNA noise bins **(E)** Correlation between noise at mRNA level and the number of overlaps in TF binding sites **(F)** Number of overlaps between TF binding sites for genes across mRNA noise bins **(G)** Number of co-expressing regulatory TFs across mRNA noise bins **(H)** Fraction of genes showing SAGA and TFIID dominance across mRNA noise bins.
(TIF)

S7 Fig Changes in on- and off-rate parameters impact burst frequency and influence mean expression level. **(A)** Relationship between on- and off-rate parameters (λ_on_ and λ_off_ respectively) and mean expression levels in cases of regulation by single TF, two independent TFs, competitive TFs and cooperative TFs. **(B)** Variation in transcription rate over time (burst frequency) in single TF regulation **(C)** Variation in transcription rate over time (burst frequency) in case of regulation by cooperatively binding TFs. For the same values of λ_on_ and λ_off_, the genes were in always on or always off states.
(TIF)

S8 Fig Noise in case of competitive TF binding was higher compared to single TF regulation across a wide range of parameter values. Changes in mRNA and protein synthesis rates, mRNA and protein degradation rates, on- and off-rate parameters (λ_on_ and λ_off_ respectively) changed mean expression levels both in single TF and competitive TF binding, but the noise levels in competitive binding were always higher than single TF binding. Increased variation in regulatory strengths of competitive TFs led to even higher noise.
(TIF)

S9 Fig Noise in case of cooperatively binding TFs was higher compared to single TF regulation across a wide range of parameter values. **(A)** Changes in mRNA and protein synthesis rates, mRNA and protein degradation rates changed mean expression levels both in single TF and cooperative TF binding, but the noise levels in cooperative binding were always higher than single TF binding. **(B)** Noise values across a wide range of on- and off-rate parameter values (λ_on_ and λ_off_ respectively) for single TF and cooperative TF binding.
(TIF)

S10 Fig No difference in the average number of mutations in the overlapping TF binding sites between low- and high-noise genes, based on calculations both at the mRNA and protein levels. (TIF)

S1 Table List of features included in our integrated model of noise. (PDF)

S1 Source Data Source data for plotting all figures in the main text and contains the following excel files. Figure1.xlsx–Source data for Fig 1; Figure2.xlsx–Source data for Fig 2; Figure3.xlsx–Source data for Fig 3; Figure4.xlsx–Source data for Fig 4; Figure5.xlsx–Source data for Fig 5; Figure7.xlsx–Source data for Fig 7.
(ZIP)
