# Supplementary material for: Transcription factor binding process is the primary driver of noise in gene expression
Source: PLoS Genet. 2022 Dec 12;18(12):e1010535. doi: 10.1371/journal.pgen.1010535 (PMC9779669; doi:10.1371/journal.pgen.1010535)
Supplement: S1 Text — (PDF) [file pgen.1010535.s001.pdf]

S1 Text

Cooperative and Competitive TF binding caused higher noise across a wide range of model parameter values

As genes could vary in their switching rates between on and off states, mRNA and protein synthesis rates and removal rates, we further explored how changes in these parameters would impact mean expression level and noise. We first quantified how changes in switching rates impacted the mean expression level in cases of single TF, cooperative TF and competitive TF regulations (S7A Fig). This assumed significance as we aimed to perform all comparisons of noise values among these three regulation scenarios at similar mean expression levels. Changes in on and off -rates resulted in alterations in burst frequency (S7B,C Fig). Some of the combinations of on- and off-rates resulted in very low or very high burst frequency and led to situations where genes were mostly off or mostly on respectively (S7B,C Fig). These resulted in very low noise irrespective of the gene regulation mechanism. Thus, to avoid these scenarios, we carefully chose the on- and off-rate parameters so as to remain within a reasonable burst frequency range. Variations in mRNA and protein synthesis rates and degradation rates altered the mean expression levels but across all parameter ranges noise values were substantially higher in the cases of competitive and cooperative TF binding compared to single TF regulation (S8,S9A Fig). Expectedly, in case of competitive TF binding, increase in variation in regulation strength of competing TFs led to further increase in noise (S8 Fig). Changes in transcription on and off-rates also altered noise levels, but again for competitive TF binding, noise was higher compared to single TF regulation (S8 Fig). For cooperatively binding TFs, changing on and off-rates resulted in substantial divergence in mean protein level compared to single TF regulation. Therefore, we compared the noise levels across all on- and off-rates and observed higher noise in case of cooperative TF binding (S9B Fig).
