# Supplementary figures and images for: Transcription factor binding process is the primary driver of noise in gene expression
Source: PLoS Genet. 2022 Dec 12;18(12):e1010535. doi: 10.1371/journal.pgen.1010535 (PMC9779669; doi:10.1371/journal.pgen.1010535)

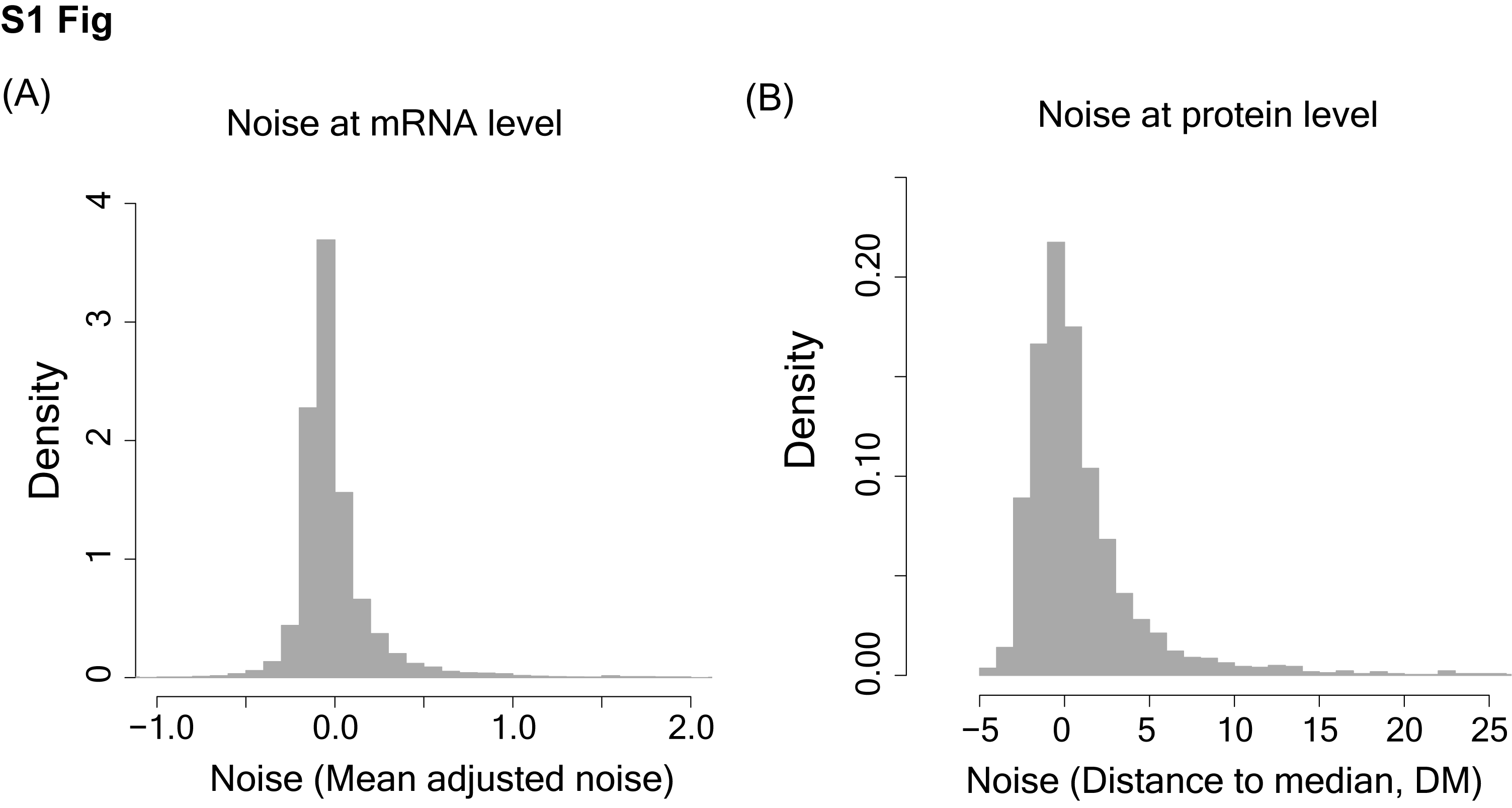

Supplement: S1 Fig — Distribution of expression noise of genes at the mRNA level (A) and at the protein level (B). (TIF) [file pgen.1010535.s002.tif]

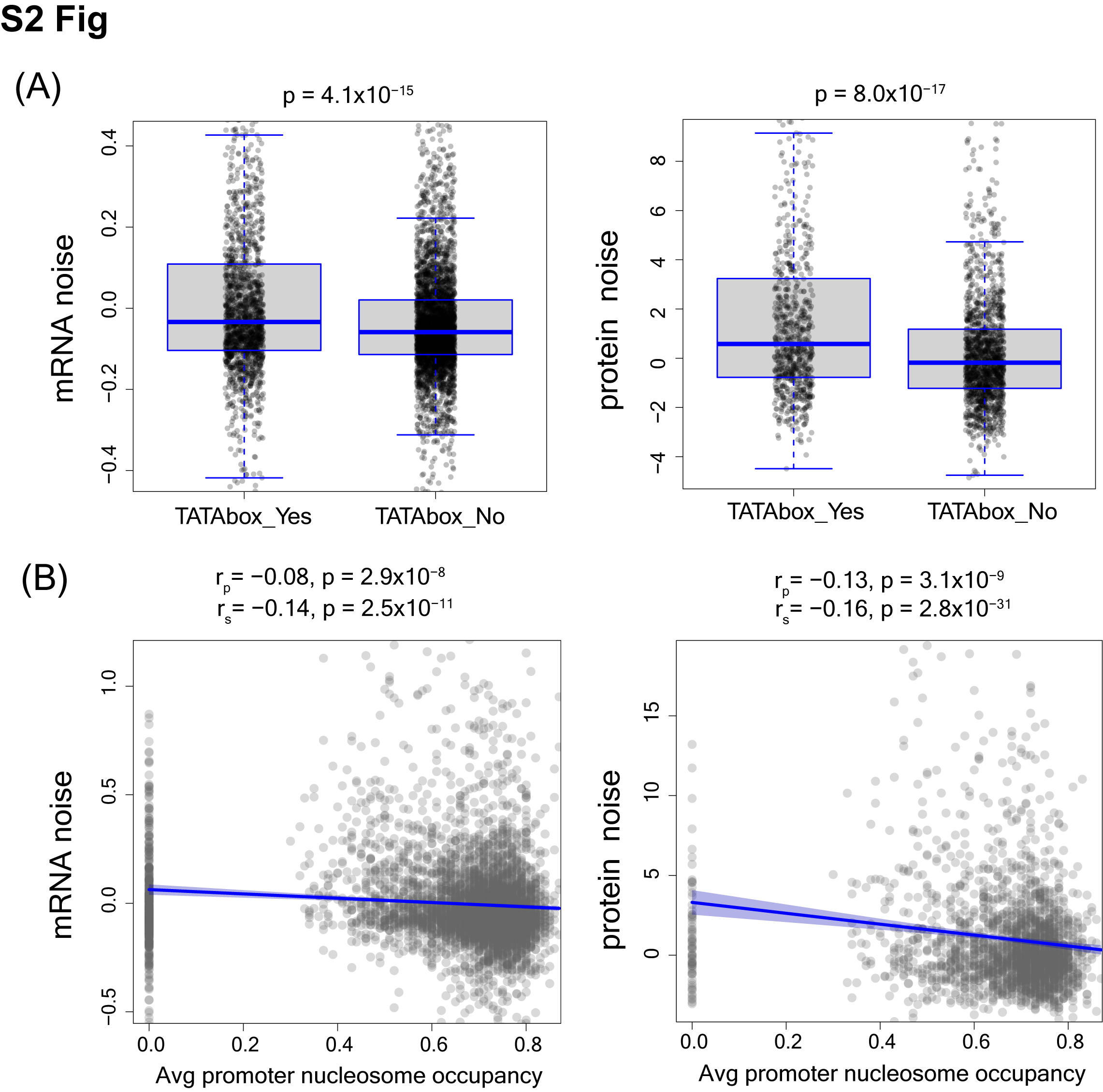

Supplement: S2 Fig — (A) Difference in expression noise of genes with and without the TATA box sequence in the promoter, calculated at the mRNA as well as the protein level (B) Correlation between noise and average promoter nucleosome occupancy. (TIF) [file pgen.1010535.s003.tif]

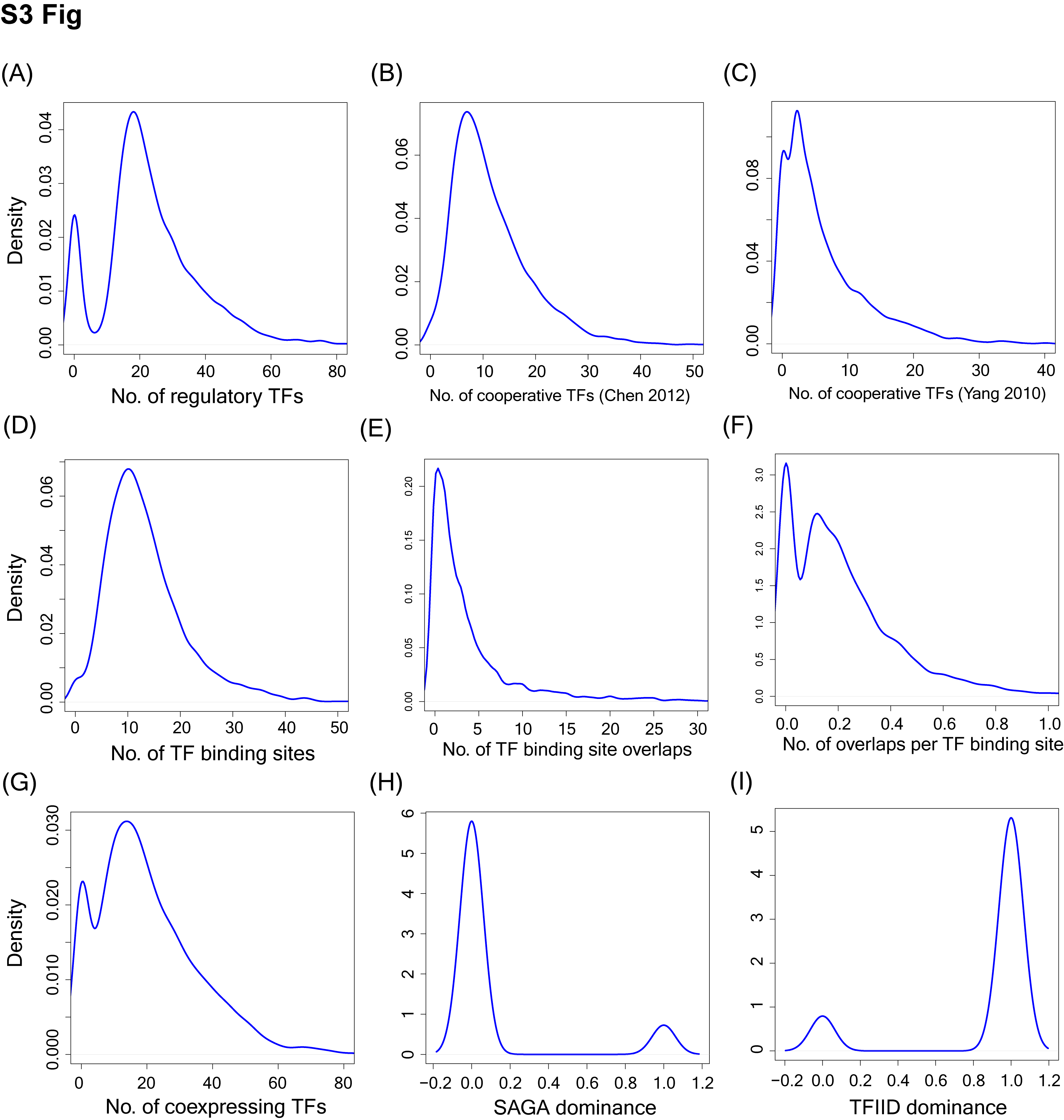

Supplement: S3 Fig — Plots showing distributions of (A) number of regulatory TFs, (B-C) number of cooperative TFs [69,70], (D) number of TF binding sites, (E) number of TF binding site overlaps, (F) number of overlaps per TF binding site, (G) number of TFs showing positive expression correlation among themselves, (H) number of genes with SAGA dominance in the promoter, and (I) number of genes with TFIID dominance in the promoter. (TIF) [file pgen.1010535.s004.tif]

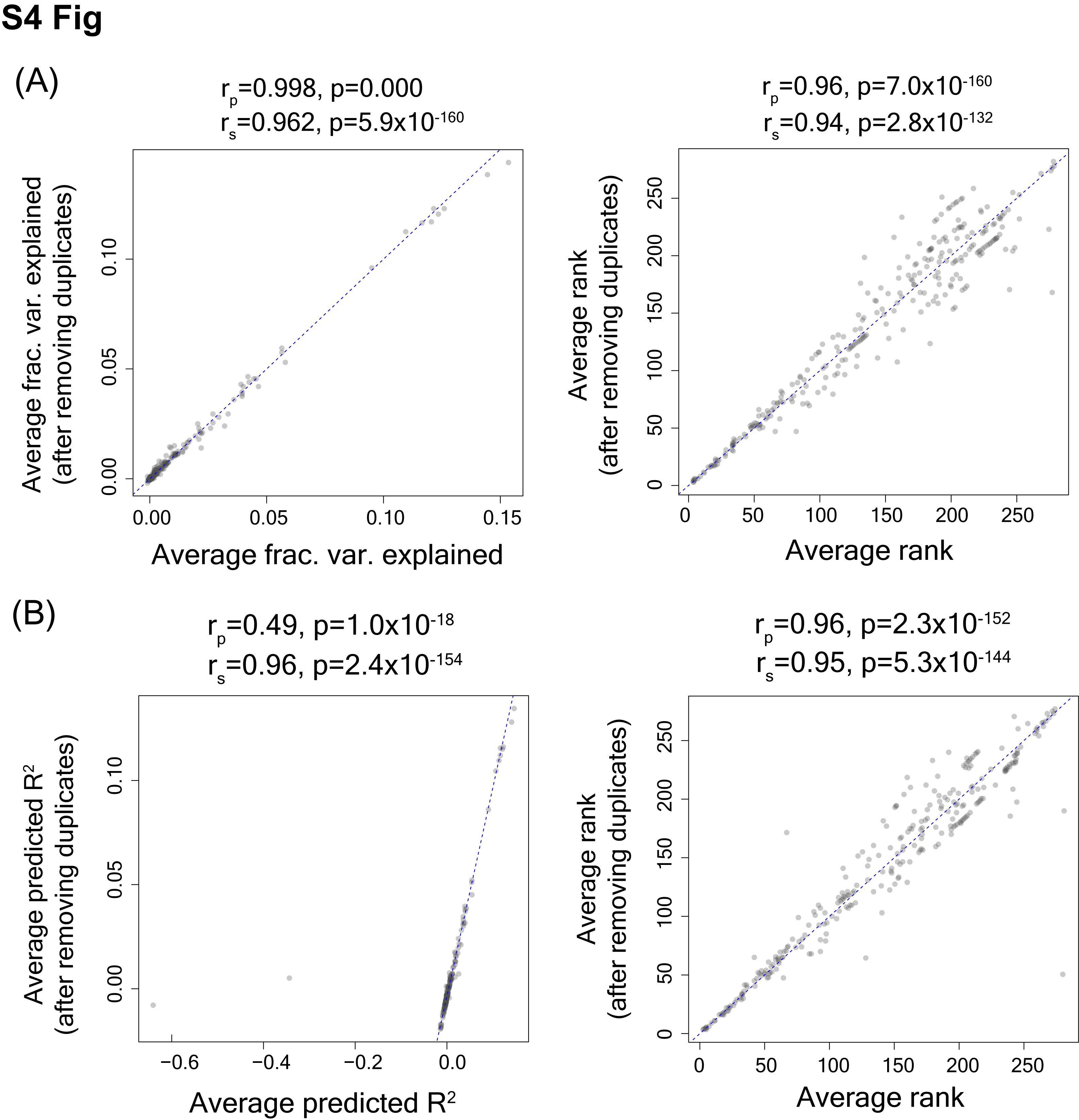

Supplement: S4 Fig — (A) Correlation between average fraction variation explained and the average rank for all features with and without the duplicate genes in the data. Average fraction variation explained and average rank for a feature were calculated by taking average of values in mRNA and protein noise data. (B) Correlation between average predicted R2 values and the corresponding average rank with and without the duplicate genes in the data. (TIF) [file pgen.1010535.s005.tif]

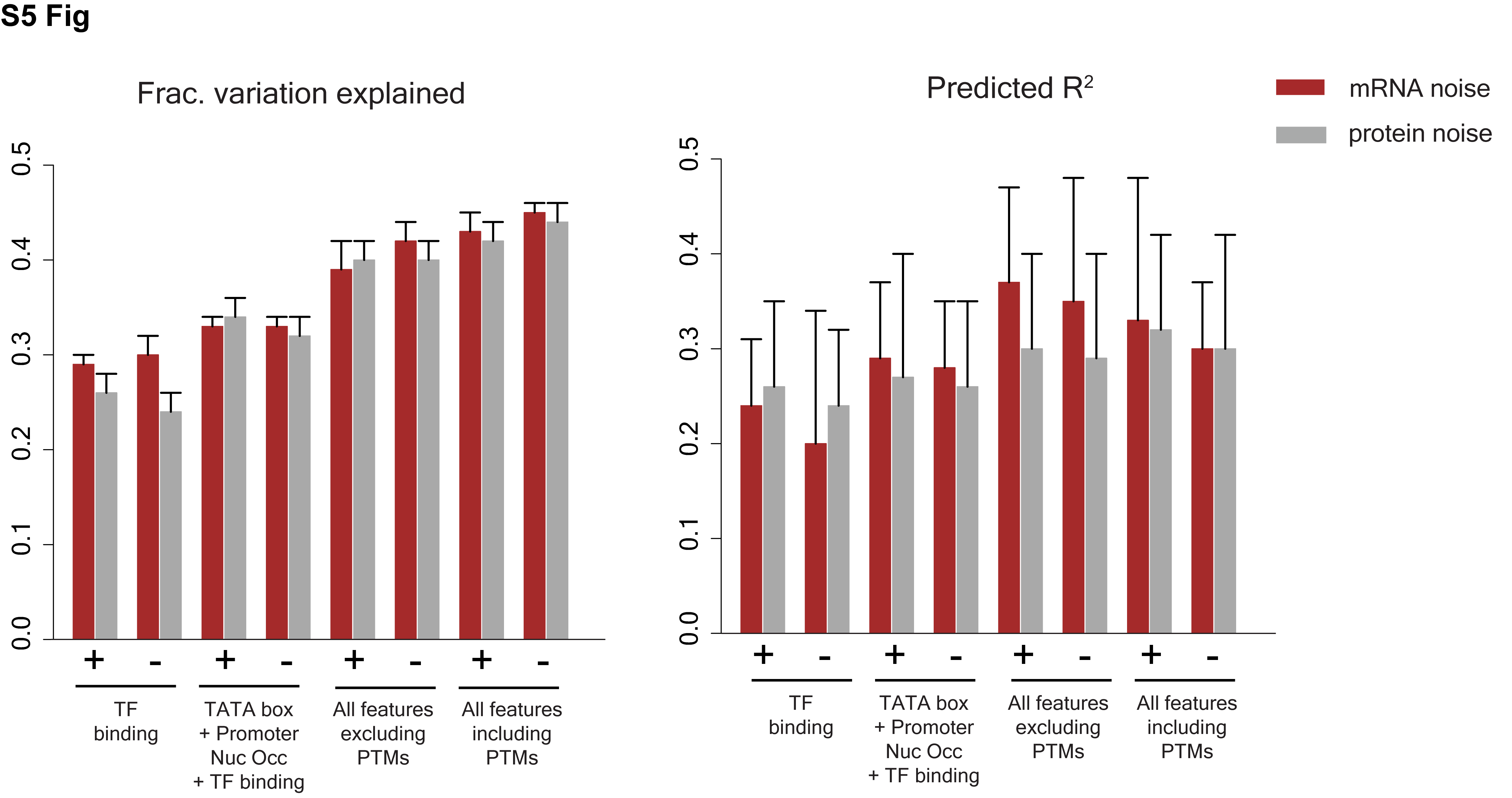

Supplement: S5 Fig — Fraction of variation explained and predictive ability of features associated with TF binding activity, combination of TF binding activity with other features, combination of all features excluding PTMs and combination of all features including PTMs. The ‘+’ and ‘-’ signs denote the datasets used in analysis, with ‘+’ indicating the full dataset and the ‘-’ sign indicating the dataset after removal of duplicate genes. (TIF) [file pgen.1010535.s006.tif]

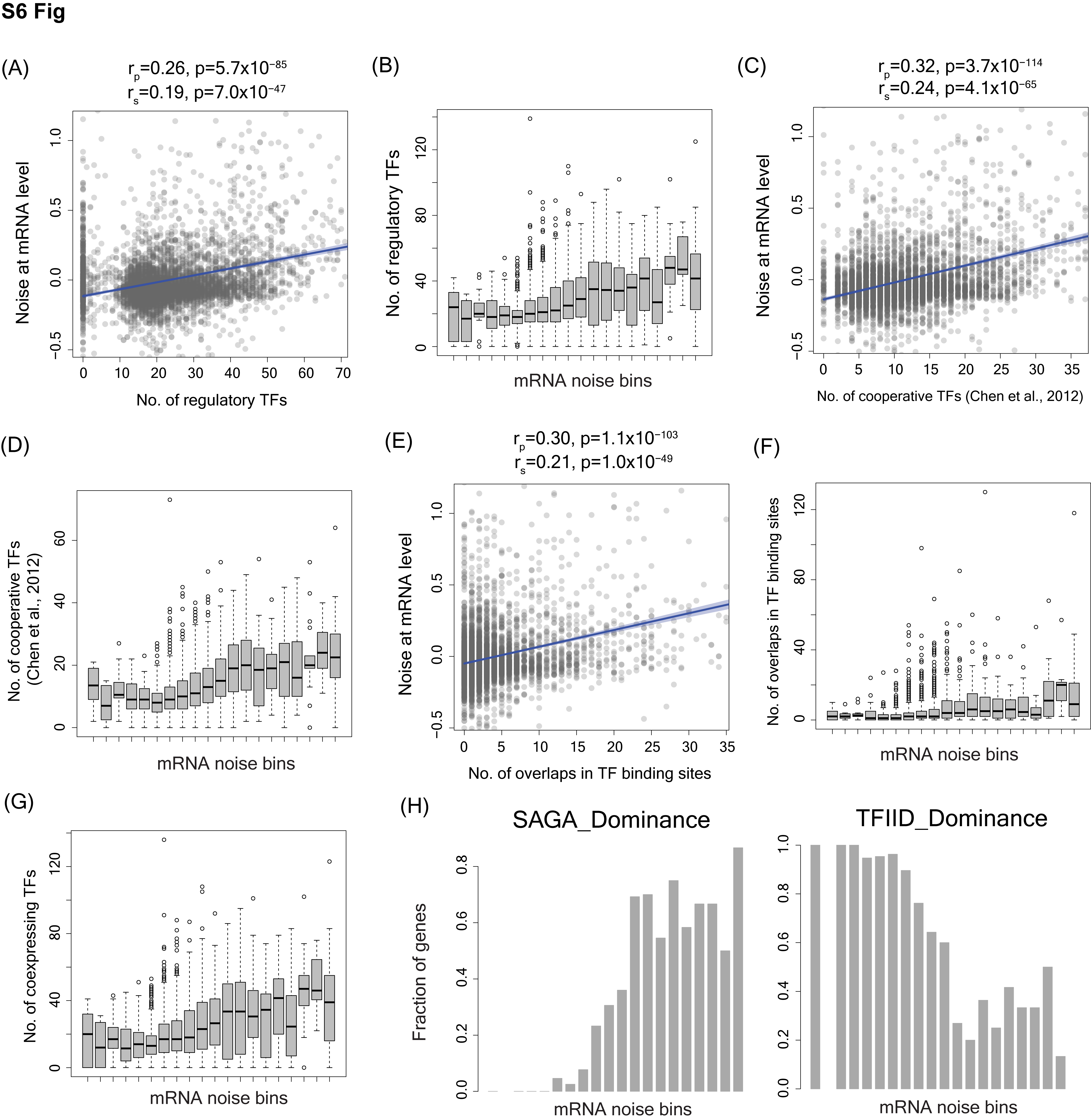

Supplement: S6 Fig — (A) Correlation between noise at mRNA level and the number of regulatory TFs (B) Number of regulatory TFs of genes across different mRNA noise bins (C) Correlation between noise at mRNA level and the number of cooperative TFs [70] (D) Number of cooperative TFs of genes across mRNA noise bins (E) Correlation between noise at mRNA level and the number of overlaps in TF binding sites (F) Number of overlaps between TF binding sites for genes across mRNA noise bins (G) Number of co-expressing regulatory TFs across mRNA noise bins (H) Fraction of genes showing SAGA and TFIID dominance across mRNA noise bins. (TIF) [file pgen.1010535.s007.tif]

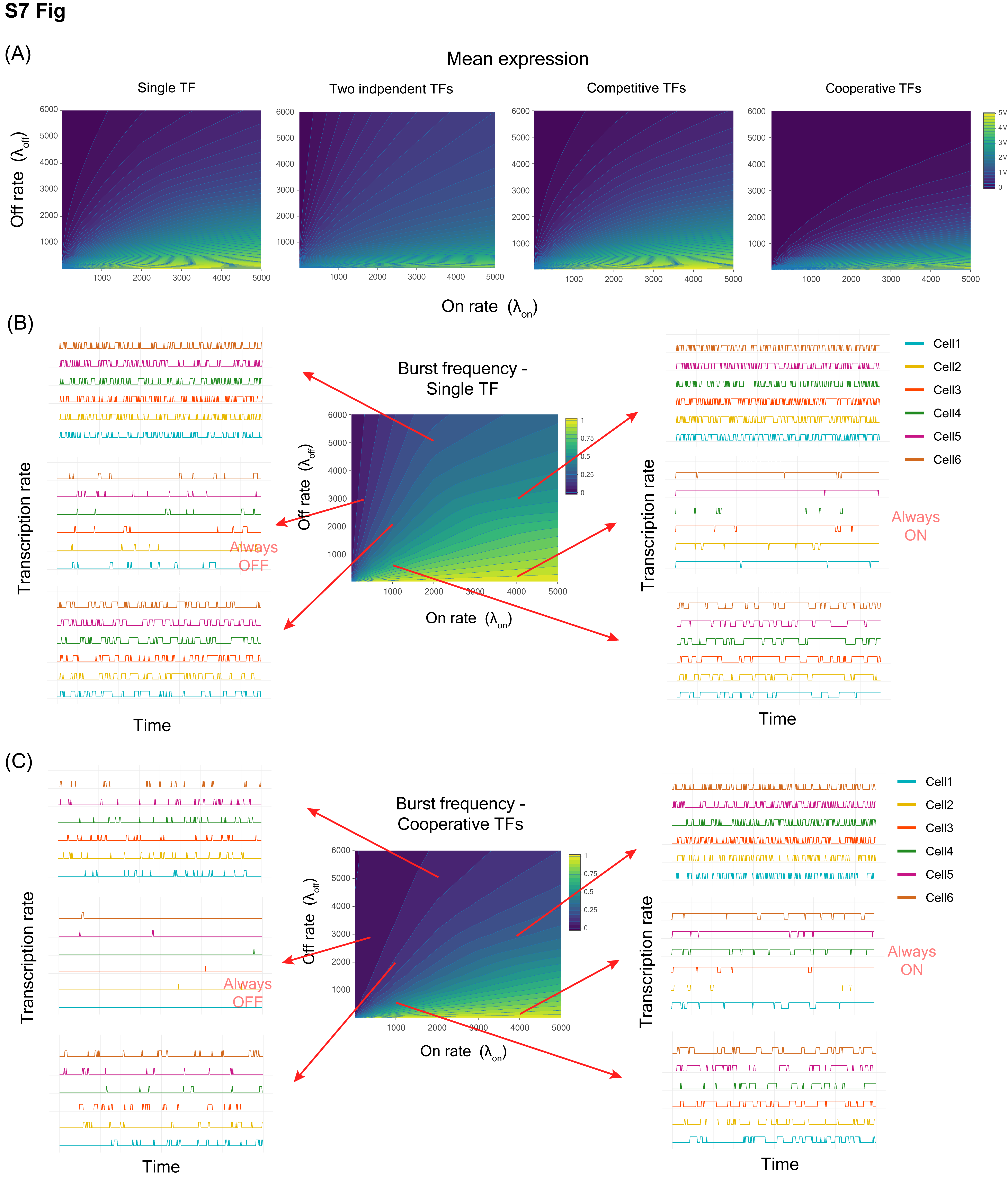

Supplement: S7 Fig — (A) Relationship between on- and off-rate parameters (λon and λoff respectively) and mean expression levels in cases of regulation by single TF, two independent TFs, competitive TFs and cooperative TFs. (B) Variation in transcription rate over time (burst frequency) in single TF regulation (C) Variation in transcription rate over time (burst frequency) in case of regulation by cooperatively binding TFs. For the same values of λon and λoff, the genes were in always on or always off states. (TIF) [file pgen.1010535.s008.tif]

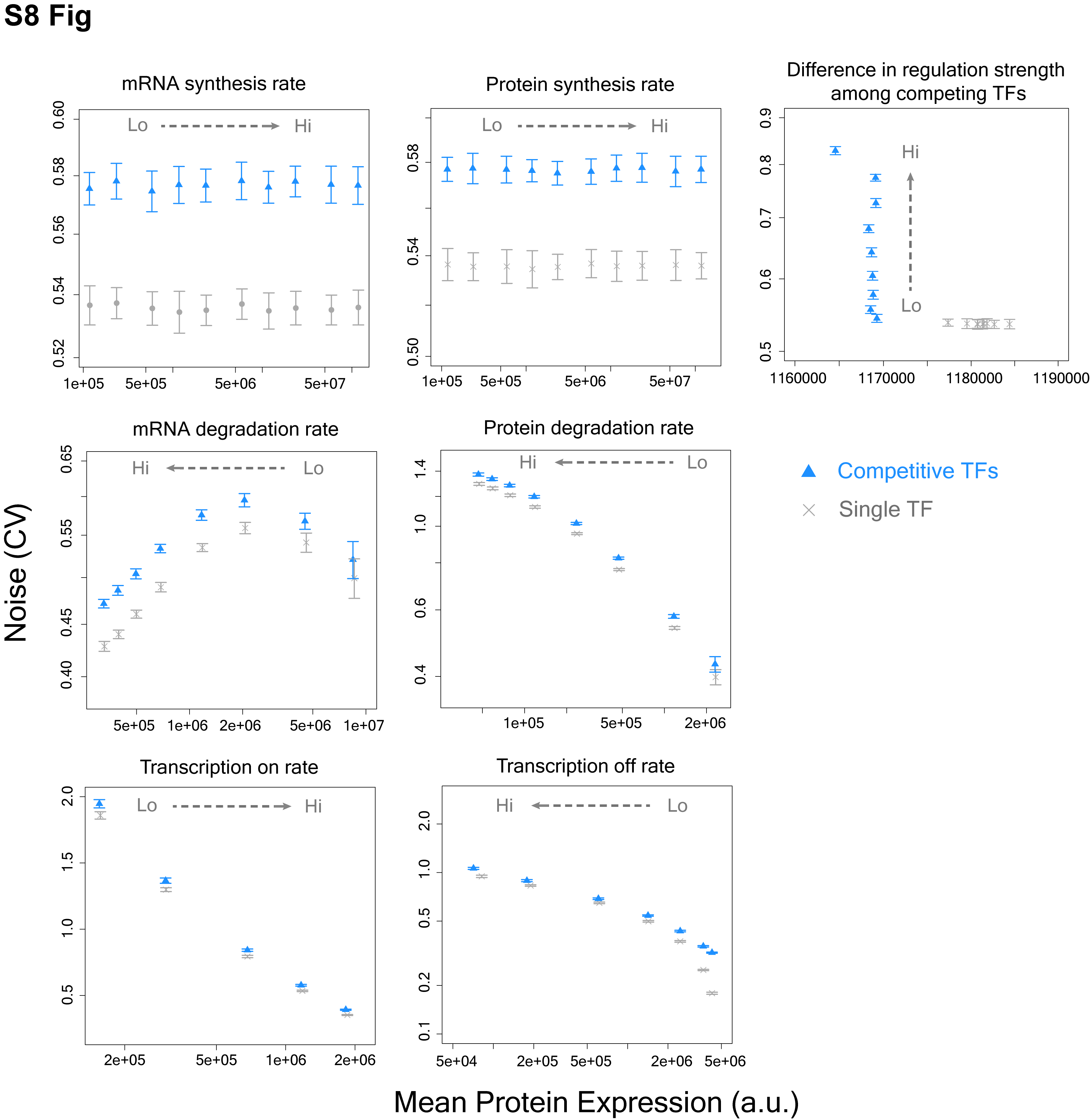

Supplement: S8 Fig — Changes in mRNA and protein synthesis rates, mRNA and protein degradation rates, on- and off-rate parameters (λon and λoff respectively) changed mean expression levels both in single TF and competitive TF binding, but the noise levels in competitive binding were always higher than single TF binding. Increased variation in regulatory strengths of competitive TFs led to even higher noise. (TIF) [file pgen.1010535.s009.tif]

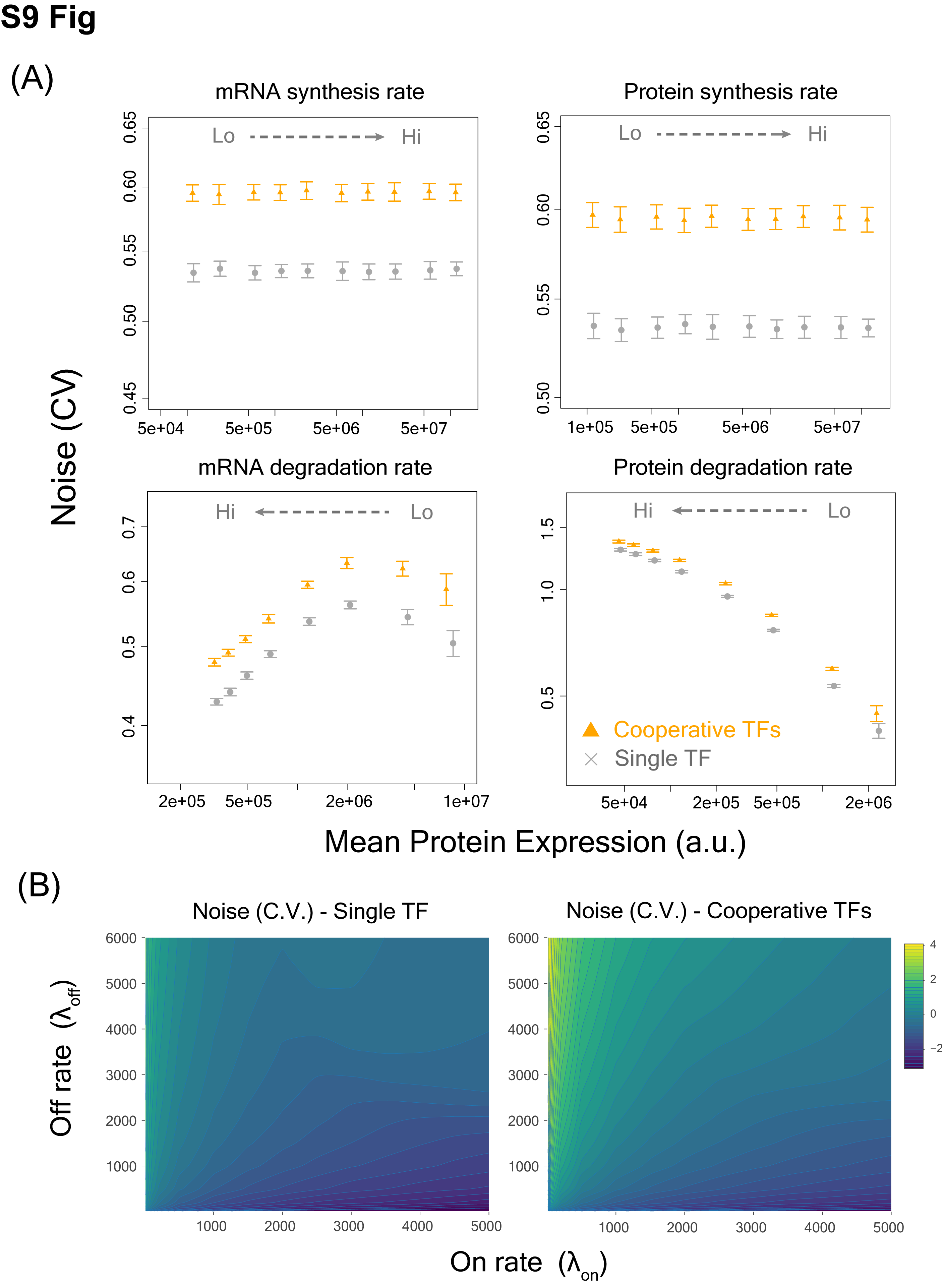

Supplement: S9 Fig — (A) Changes in mRNA and protein synthesis rates, mRNA and protein degradation rates changed mean expression levels both in single TF and cooperative TF binding, but the noise levels in cooperative binding were always higher than single TF binding. (B) Noise values across a wide range of on- and off-rate parameter values (λon and λoff respectively) for single TF and cooperative TF binding. (TIF) [file pgen.1010535.s010.tif]

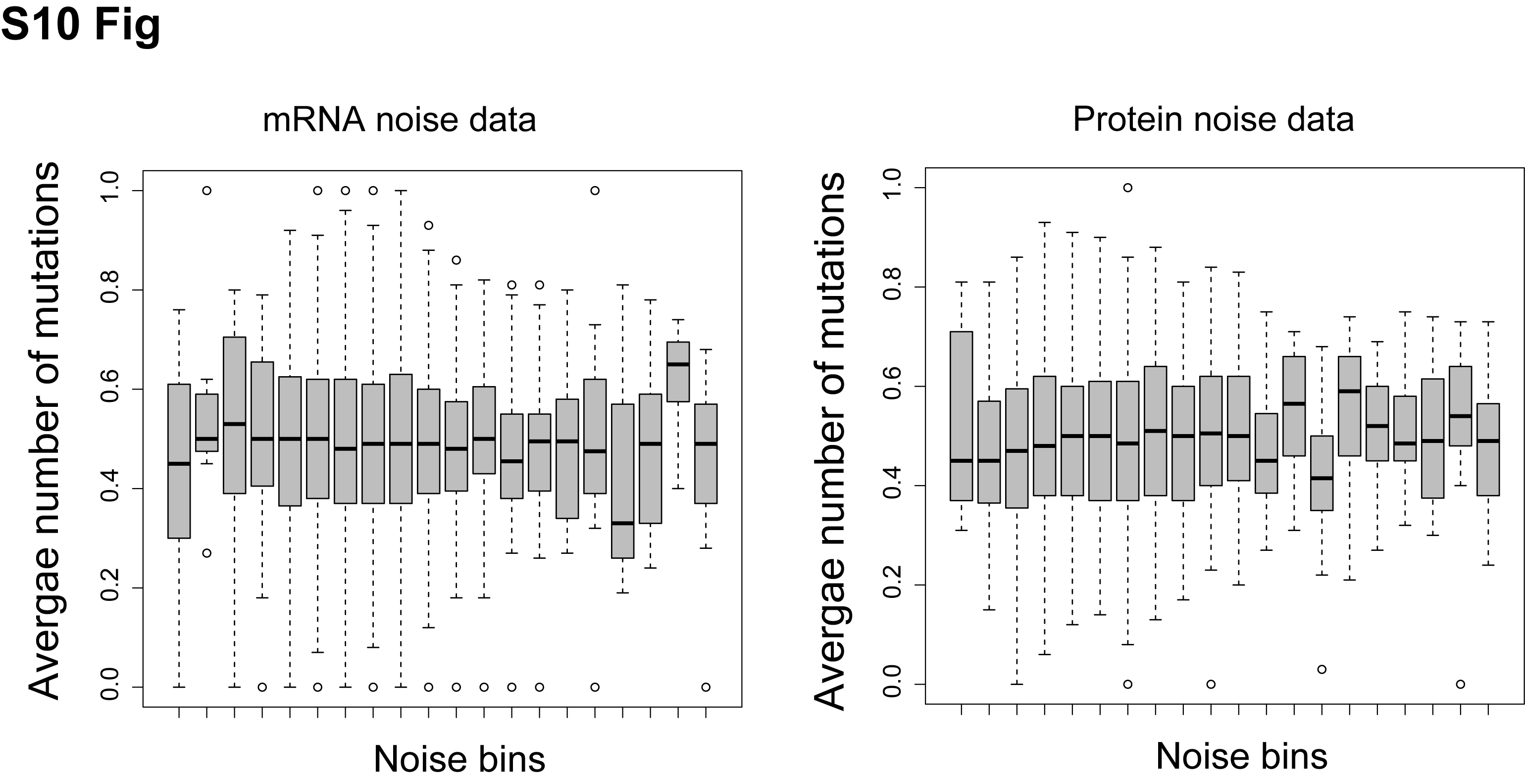

Supplement: S10 Fig — (TIF) [file pgen.1010535.s011.tif]
