# Supplementary material for: Transcription factor binding process is the primary driver of noise in gene expression
Source: PLoS Genet. 2022 Dec 12;18(12):e1010535. doi: 10.1371/journal.pgen.1010535 (PMC9779669; doi:10.1371/journal.pgen.1010535)
Supplement: S1 Table — (PDF) [file pgen.1010535.s012.pdf]

S1 Table - List of features included in our integrated model of noise

Feature name	Description	References
STRE_elem	Presence/Absence of the Stress Response Element in the promoter	[1]
TATAbox	presence/Absence of the TATAbox sequence in the promoter	[2]
ConsensClustNum	Number of consensus clusters of transcription start sites	[3]
ClosestTSS	Closest transcription start site to the coding region	
SpreadTSS	Spread of potential transcription start sites	
MedPromShapeScore	Median promoter shape score	
tAI_full	tRNA adaptation index for the full gene	[4]
tAI_f5	tRNA adaptation index for the first 5 codons	
tAI_f10	tRNA adaptation index for the first 10 codons	
tAI_f15	tRNA adaptation index for the first 15 codons	
tAI_f20	tRNA adaptation index for the first 20 codons	
tAI_f25	tRNA adaptation index for the first 25 codons	
tAI_f30	tRNA adaptation index for the first 30 codons	
tAI_f40	tRNA adaptation index for the first 40 codons	
tAI_f50	tRNA adaptation index for the first 50 codons	
NumPromNucOcc	Number of sites in the promoter occupied by nucleosomes	[5]
AvPromNucOcc	Average absolute nucleosome occupancy level per nucleosome bound site in the promoter	
LenAvgPromNucOcc	Average length of nucleosome occupancy in the promoter	
NumGeneNucOcc	Number of sites in the genebody occupied by nucleosomes	
AvGeneNucOcc	Average absolute nucleosome occupancy level per nucleosome bound site in the genebody	
N_PrNO50	Number of sites upto 50 bp upstream of the start codon occupied by nucleosomes	
N_PrNO100	Number of sites between 50 bp and 100bp upstream of the start codon occupied by nucleosomes	
N_PrNO150	Number of sites between 100 bp and 150bp upstream of the start codon occupied by nucleosomes	
N_PrNO200	Number of sites between 150 bp and 200bp upstream of the start codon occupied by nucleosomes	
N_PrNO300	Number of sites between 200 bp and 300bp upstream of the start codon occupied by nucleosomes	
N_PrNO400	Number of sites between 300 bp and 400bp upstream of the start codon occupied by nucleosomes	
N_PrNO500	Number of sites between 400 bp and 500bp upstream of the start codon occupied by nucleosomes	
N_PrNO600	Number of sites between 500 bp and 600bp upstream of the start codon occupied by nucleosomes	
N_PrNO700	Number of sites between 600 bp and 700bp upstream of the start codon occupied by nucleosomes	
N_PrNO800	Number of sites between 700 bp and 800bp upstream of the start codon occupied by nucleosomes	
N_PrNO900	Number of sites between 800 bp and 900bp upstream of the start codon occupied by nucleosomes	
N_PrNO1000	Number of sites between 900 bp and 1000bp upstream of the start codon occupied by nucleosomes	
V_PrNO50	Level of absolute nucleosome occupancy in the region upto 50bp upstream of the start codon	
V_PrNO100	Level of absolute nucleosome occupancy in the region between 50bp and 100bp upstream of the start codon	
V_PrNO150	Level of absolute nucleosome occupancy in the region between 100bp and 150bp upstream of the start codon	
V_PrNO200	Level of absolute nucleosome occupancy in the region between 150bp and 200bp upstream of the start codon	
V_PrNO300	Level of absolute nucleosome occupancy in the region between 200bp and 300bp upstream of the start codon	

V_PrNO400	Level of absolute nucleosome occupancy in the region between 300bp and 400bp upstream of the start codon	
V_PrNO500	Level of absolute nucleosome occupancy in the region between 400bp and 500bp upstream of the start codon	
V_PrNO600	Level of absolute nucleosome occupancy in the region between 500bp and 600bp upstream of the start codon	
V_PrNO700	Level of absolute nucleosome occupancy in the region between 600bp and 700bp upstream of the start codon	
V_PrNO800	Level of absolute nucleosome occupancy in the region between 700bp and 800bp upstream of the start codon	
V_PrNO900	Level of absolute nucleosome occupancy in the region between 800bp and 900bp upstream of the start codon	
V_PrNO1000	Level of absolute nucleosome occupancy in the region between 900bp and 1000bp upstream of the start codon	
NumGb_H3	Number of sites in the genebody occupied by H3	[6]
Gb_H3	Level of H3 in the genebody	
NumGb_H4	Number of sites in the genebody occupied by H4	
Gb_H4	Level of H4 in the genebody	
NumGb_H3K9ac_vsH3	Number of sites in the genebody showing H3K9ac modification	
Gb_H3K9ac_vsH3	Level of H3K9ac modification in the genebody	
NumGb_H3K14ac_vsH3	Number of sites in the genebody showing H3K14ac modification	
Gb_H3K14ac_vsH3	Level of H3K14ac modification in the genebody	
NumGb_H4ac_vsH3	Number of sites in the genebody showing H4ac modification	
Gb_H4ac_vsH3	Level of H4ac modification in the genebody	
NumGb_H3K4me1_vsH3	Number of sites in the genebody showing H3K4me1 modification	
Gb_H3K4me1_vsH3	Level of H3K4me1 modification in the genebody	
NumGb_H3K4me2_vsH3	Number of sites in the genebody showing H3K4me2 modification	
Gb_H3K4me2_vsH3	Level of H3K4me2 modification in the genebody	
NumGb_H3K4me3_vsH3	Number of sites in the genebody showing H3K4me3 modification	
Gb_H3K4me3_vsH3	Level of H3K4me3 modification in the genebody	
NumGb_H3K36me3_vsH3	Number of sites in the genebody showing H3K36me3 modification	
Gb_H3K36me3_vsH3	Level of H3K36me3 modification in the genebody	
NumGb_H3K79me3_vsH3	Number of sites in the genebody showing H3K79me3 modification	
Gb_H3K79me3_vsH3	Level of H3K79me3 modification in the genebody	
NumGb_ESA1	Number of sites in the genebody showing ESA binding	
Gb_ESA1	Level of ESA1 binding in the genebody	
NumGb_GCN5	Number of sites in the genebody showing GCN5 binding	
Gb_GCN5	Level of GCN5 binding in the genebody	
NumGb_GCN4.AA	Number of sites in the genebody showing GCN4 binding	
Gb_GCN4.AA	Level of GCN4 binding in the genebody	
NumProm_H3	Number of sites in the promoter occupied by H3	
Prom_H3	Level of H3 in the promoter	
NumProm_H4	Number of sites in the promoter occupied by H4	
Prom_H4	Level of H4 in the promoter	
NumProm_H3K9ac_vsH3	Number of sites in the promoter showing H3K9ac modification	
Prom_H3K9ac_vsH3	Level of H3K9ac modification in the promoter	
NumProm_H3K14ac_vsH3	Number of sites in the promoter showing H3K14ac modification	
Prom_H3K14ac_vsH3	Level of H3K14ac modification in the promoter	
NumProm_H4ac_vsH3	Number of sites in the promoter showing H4ac modification	
Prom_H4ac_vsH3	Level of H4ac modification in the promoter	
NumProm_H3K4me1_vsH3	Number of sites in the promoter showing H3K4me1 modification	
Prom_H3K4me1_vsH3	Level of H3K4me1 modification in the promoter	
NumProm_H3K4me2_vsH3	Number of sites in the promoter showing H3K4me2 modification	
Prom_H3K4me2_vsH3	Level of H3K4me2 modification in the promoter	

NumProm_H3K4me3_vsH3	Number of sites in the promoter showing H3K4me3 modification	
Prom_H3K4me3_vsH3	Level of H3K4me3 modification in the promoter	
NumProm_H3K36me3_vsH3	Number of sites in the promoter showing H3K36me3 modification	
Prom_H3K36me3_vsH3	Level of H3K36me3 modification in the promoter	
NumProm_H3K79me3_vsH3	Number of sites in the promoter showing H3K79me3 modification	
Prom_H3K79me3_vsH3	Level of H3K79me3 modification in the promoter	
NumProm_ESA1	Number of sites in the promoter showing ESA1 binding	
Prom_ESA1	Level of ESA1 binding in the promoter	
NumProm_GCN5	Number of sites in the promoter showing GCN5 binding	
Prom_GCN5	Level of GCN5 binding in the promoter	
NumProm_GCN4.AA	Number of sites in the promoter showing GCN4 binding	
Prom_GCN4.AA	Level of GCN4 binding in the promoter	
DionScience2007_Gene_G1Lambda	H3 turnover rate in the coding region of G1 arrested yeast	[7]
DionScience2007_Gene_G1Lambda_Zscore	H3 turnover rate in the coding region of G1 arrested yeast (Z-score calculated)	
DionScience2007_Prom_G1Lambda	H3 turnover rate in the promoter region of G1 arrested yeast	
DionScience2007_Prom_G1Lambda_Zscore	H3 turnover rate in the promoter region of G1 arrested yeast (Z-score calculated)	
DionScience2007_Gene_H3Occ	H3 occupancy in the coding region	
DionScience2007_Gene_NucOcc	Nucleosome occupancy in the coding region	
DionScience2007_Prom_H3Occ	H3 occupancy in the promoter region	
DionScience2007_Prom_NucOcc	Nucleosome occupancy in the promoter region	
DionScience2007_Gene_PolII_t0	RNA pol II occupancy in the coding region at t=0	
DionScience2007_Gene_PolII_t60	RNA pol II occupancy in the coding region at t=60 mins	
DionScience2007_Prom_PolIII_t0	RNA pol II occupancy in the promoter region at t=0	
DionScience2007_Prom_PolIII_t60	RNA pol II occupancy in the promoter region at t=60 mins	
Sun2012_mRNA_Synth_rate	mRNA synthesis rate	[8]
Sun2012_mRNA_Decay_rate	mRNA decay rate	
mRNA_PARS1	mRNA secondary structure PARS score of the first codon	[9]
mRNA_PARS3	mRNA secondary structure PARS score of the first three codons	
mRNA_PARS5	mRNA secondary structure PARS score of the first five codons	
mRNA_PARS10	mRNA secondary structure PARS score of the first ten codons	
mRNA_PARS15	mRNA secondary structure PARS score of the first fifteen codons	
mRNA_PARS20	mRNA secondary structure PARS score of the first twenty codons	
mRNA_PARS25	mRNA secondary structure PARS score of the first twenty-five codons	
mRNA_PARS50	mRNA secondary structure PARS score of the first fifty codons	
mRNA_HL_Mins	mRNA half-life in minutes	[10]
protein_HL_Mins	protein half-life in minutes	[11]
Phosphorylation	Number of residues in the protein with phosphorylation	[12]
Methylation	Number of residues in the protein with methylation	
Acetylation	Number of residues in the protein with acetylation	
Ubiquitination	Number of residues in the protein with ubiquitination	
Succinylation	Number of residues in the protein with succinylation	
Oxidation	Number of residues in the protein showing oxidation	
Nitration	Number of residues in the protein showing nitration	
NtAcetylation	Number of residues in the protein with N-terminal acrtylation	
Glycosylation	Number of residues in the protein with glycosylation	
Ca	Number of calcium binding sites in the protein	
Disulfide	Number of residues in the protein showing disulfide bond formation	
Lipidation	Number of residues in the protein with lipidation	
ActiveSite	Number of residues in the active site of the protein	
Sumoylation	Number of residues in the protein with SUMOylation	

whetherTF	Whether the gene is a TF (Yes/No)	[6,13-16]
Num_RegTF_YeastractYT	Number of regulatory TFs (from Yeastract data)	
MedExp_TFYT	Median expression of regulatory TFs (Yeastract data)	
MedDM_SD_TFYT	Median noise of regulatory TFs (Yeastract data)	
MedPosDM_SD_TFYT	Median positive noise (DM values) of regulatory TFs (Yeastract data)	
MedNegDM_SD_TFYT	Median negative noise (DM values) of regulatory TFs (Yeastract data)	
PercNegDM_TFYT	Percentage of TFs showing negative noise (DM) values	
PercPosDM_TFYT	Percentage of TFs showing positive noise (DM) values	
minDM_TFYT	Minimum noise (DM) value	
maxDM_TFYT	Maximum noise (DM) value	
PosCorTF_YT	Number of TFs showing positive expression correlation with the target gene	
NegCorTF_YT	Number of TFs showing negative expression correlation with the target gene	
PosCorTF.NegCorTF_YT	Ratio of the number of TFs showing positive expression correlation to the number of TFs showing negative expression correlation with the target gene	
PercPosCorTF_YT	Percentage of TFs showing positive expression correlation with the target gene	
PercNegCorTF_YT	Percentage of TFs showing negative expression correlation with the target gene	
Both_PosCorr_NegCorrTF_YT	Whether the gene has positively and negatively correlated TFs (Yes/No)	
NoisePosCorTF_YT	Noise (DM) value of TFs showing positive expression correlation with the target gene	
NoiseNegCorTF_YT	Noise (DM) value of TFs showing negative expression correlation with the target gene	
MeanStrPosCorTF	Mean regulation strength of positively correlated TFs	
SdStrPosCorTF	Sd regulation strength of positively correlated TFs	
MeanStrNegCorTF	Mean regulation strength of negatively correlated TFs	
SdStrNegCorTF	Sd regulation strength of negatively correlated TFs	
MeanCorPosCorTF	Mean correlation value of TFs showing positive expression correlation with the target gene	
SdCorPosCorTF	Sd correlation value of TFs showing positive expression correlation with the target gene	
MeanCorNegCorTF	Mean correlation value of TFs showing negative expression correlation with the target gene	
SdCorNegCorTF	Sd correlation value of TFs showing negative expression correlation with the target gene	
NumPosCor_withinTFs	Number of TFs showing positive expression correlation with other TFs regulating the same target gene	
NumNegCor_withinTFs	Number of TFs showing negative expression correlation with other TFs regulating the same target gene	
PercPosCor_withinTFs	Percentage of TFs showing positive expression correlation with other TFs regulating the same target gene	
PercNegCor_withinTFs	Percentage of TFs showing negative expression correlation with other TFs regulating the same target gene	
PosCor.NegCor_withinTFs	Ratio of the number of TFs showing positive expression correlation with other regulating TFs of a gene to the number of TFs showing negative expression correlation with other TFs of a gene	
PercPosCorWN_OVsites	Percentage of TFs showing positive expression correlation with other TFs regulating the same target gene and binding to overlapping binding sites in the promoter	
PercNegCorWN_OVsites	Percentage of TFs showing negative expression correlation with other TFs regulating the same target gene and binding to overlapping binding sites in the promoter	
AvgMut	Average number of mutations in the TF binding motifs in the promoter region	

N_TFSites100	Number of TF binding sites upto 100bp upstream region of the start codon
N_TFSites200	Number of TF binding sites within 100bp and 200bp upstream region of the start codon
N_TFSites300	Number of TF binding sites within 200bp and 300bp upstream region of the start codon
N_TFSites400	Number of TF binding sites within 300bp and 400bp upstream region of the start codon
N_TFSites500	Number of TF binding sites within 400bp and 500bp upstream region of the start codon
N_TFSites600	Number of TF binding sites within 500bp and 600bp upstream region of the start codon
N_TFSites700	Number of TF binding sites within 600bp and 700bp upstream region of the start codon
N_TFSites800	Number of TF binding sites within 700bp and 800bp upstream region of the start codon
N_TFSites900	Number of TF binding sites within 800bp and 900bp upstream region of the start codon
N_TFSites1000	Number of TF binding sites within 900bp and 1000bp upstream region of the start codon
ExpTF100	Mean expression of TFs binding upto 100bp upstream region of the start codon
NoiseTF100	Expression noise of TFs binding upto 100bp upstream region of the start codon
ExpTF200	Mean expression of TFs binding between 100bp and 200bp upstream region of the start codon
NoiseTF200	Expression noise of TFs binding between 100bp and 200bp upstream region of the start codon
ExpTF300	Mean expression of TFs binding between 200bp and 300bp upstream region of the start codon
NoiseTF300	Expression noise of TFs binding between 200bp and 300bp upstream region of the start codon
ExpTF400	Mean expression of TFs binding between 300bp and 400bp upstream region of the start codon
NoiseTF400	Expression noise of TFs binding between 300bp and 400bp upstream region of the start codon
ExpTF500	Mean expression of TFs binding between 400bp and 500bp upstream region of the start codon
NoiseTF500	Expression noise of TFs binding between 400bp and 500bp upstream region of the start codon
ExpTF600	Mean expression of TFs binding between 500bp and 600bp upstream region of the start codon
NoiseTF600	Expression noise of TFs binding between 500bp and 600bp upstream region of the start codon
ExpTF700	Mean expression of TFs binding between 600bp and 700bp upstream region of the start codon
NoiseTF700	Expression noise of TFs binding between 600bp and 700bp upstream region of the start codon
ExpTF800	Mean expression of TFs binding between 700bp and 800bp upstream region of the start codon
NoiseTF800	Expression noise of TFs binding between 700bp and 800bp upstream region of the start codon
ExpTF900	Mean expression of TFs binding between 800bp and 900bp upstream region of the start codon
NoiseTF900	Expression noise of TFs binding between 800bp and 900bp upstream region of the start codon
ExpTF1000	Mean expression of TFs binding between 900bp and 1000bp upstream region of the start codon
NoiseTF1000	Expression noise of TFs binding between 900bp and 1000bp upstream region of the start codon
PercTFsiteNuc	Percentage of TF sites showing nucelosome occupancy
PercTFsiteHistMod	Percentage of TF sites with histone modifications

AvgTFsiteNucOcc	Average TF site nucleosome occupancy level
AvgTFsiteHist	Average TF site histone level
AvgTFsiteMod	Average TF site histone modifications
AvgTFsiteAsoc	Average level of associated regulators and modifiers (GCN4,GCN5,ESA1) in TF binding sites
PercOfPromNuc	Percentage of the total promoter nucleosome occupancy level observed in the TF binding sites
PercOfPromHist	Percentage of the total promoter histone level observed in the TF binding sites
PercOfPromMod	Percentage of the total promoter histone modifications observed in the TF binding sites
PercOfPromAsoc	Percentage of the total promoter associated regulators and modifiers observed in the TF binding sites
TFsiteH3	H3 level in TF binding sites
TFsiteH4	H4 level in TF binding sites
TFsiteH3K9ac_vsH3	Level of H3K9ac modifications in TF binding sites
TFsiteH3K14ac_vsH3	Level of H3K14ac modifications in TF binding sites
TFsiteH4ac_vsH3	Level of H4ac modifications in TF binding sites
TFsiteH3K4me1_vsH3	Level of H3K4me1 modifications in TF binding sites
TFsiteH3K4me2_vsH3	Level of H3K4me2 modifications in TF binding sites
TFsiteH3K4me3_vsH3	Level of H3K4me3 modifications in TF binding sites
TFsiteH3K36me3_vsH3	Level of H3K36me3 modifications in TF binding sites
TFsiteH3K79me3_vsH3	Level of H3K79me3 modifications in TF binding sites
TFsiteESA1	Level of ESA1 in TF binding sites
TFsiteGCN5	Level of GCN5 in TF binding sites
TFsiteGCN4.AA	Level of GCN4 in TF binding sites
PercOfPromH3	Percentage of the total promoter H3 level observed in the TF binding sites
PercOfPromH4	Percentage of the total promoter H4 level observed in the TF binding sites
PercOfPromH3K9ac_vsH3	Percentage of the total promoter H3K9ac level observed in the TF binding sites
PercOfPromH3K14ac_vsH3	Percentage of the total promoter H3K14ac level observed in the TF binding sites
PercOfPromH4ac_vsH3	Percentage of the total promoter H4ac level observed in the TF binding sites
PercOfPromH3K4me1_vsH3	Percentage of the total promoter H3K4me1 level observed in the TF binding sites
PercOfPromH3K4me2_vsH3	Percentage of the total promoter H3K4me2 level observed in the TF binding sites
PercOfPromH3K4me3_vsH3	Percentage of the total promoter H3K4me3 level observed in the TF binding sites
PercOfPromH3K36me3_vsH3	Percentage of the total promoter H3K36me3 level observed in the TF binding sites
PercOfPromH3K79me3_vsH3	Percentage of the total promoter H3K79me3 level observed in the TF binding sites
PercOfPromESA1	Percentage of the total promoter ESA1 level observed in the TF binding sites
PercOfPromGCN5	Percentage of the total promoter GCN5 level observed in the TF binding sites
PercOfPromGCN4.AA	Percentage of the total promoter GCN4 level observed in the TF binding sites
NumSites	Number of TF binding sites in the promoter region
NumOverlaps	Number of overlaps in TF binding sites in the promoter region
RatOverlap.Numsites	Ratio of the number of overlaps to the total number of TF binding sites
AvgOverlapLen	Average overlap length
AvgfrOverlapLen	Average fraction of TF binding site showing overlap
OV100	Percentage of binding site overlaps up to 100bp upstream region of the start codon of all overlaps in the promoter
OV200	Percentage of binding site overlaps between 100bp and 200bp upstream region of the start codon

OV300	Percentage of binding site overlaps between 200bp and 300bp upstream region of the start codon	
OV400	Percentage of binding site overlaps between 300bp and 400bp upstream region of the start codon	
OV500	Percentage of binding site overlaps between 400bp and 500bp upstream region of the start codon	
OV600	Percentage of binding site overlaps between 500bp and 600bp upstream region of the start codon	
OV700	Percentage of binding site overlaps between 600bp and 700bp upstream region of the start codon	
OV800	Percentage of binding site overlaps between 700bp and 800bp upstream region of the start codon	
OV900	Percentage of binding site overlaps between 800bp and 900bp upstream region of the start codon	
OV1000	Percentage of binding site overlaps between 900bp and 1000bp upstream region of the start codon	
PercAct2_Overlap	Percentage of overlapping sites shared by two activators	
Avg_stract2_ov	Average strength of regulation of two activators binding to overlapping sites	
Df_stract2_ov	Difference in strength of regulation of two activators binding to overlapping sites	
Avg_corstr_act2_ov	Average expression correlation of two activators binding to overlapping sites with the target gene	
Df_corstr_act2_ov	Difference in expression correlation of two activators binding to overlapping sites with the target gene	
PercRep2_overlap	Percentage of overlapping sites shared by two repressors	
Avg_strrep2_ov	Average strength of regulation of two repressors binding to overlapping sites	
Df_strrep2_ov	Difference in strength of regulation of two repressors binding to overlapping sites	
Avg_corstr_rep2_ov	Average expression correlation of two repressors binding to overlapping sites with the target gene	
Df_corstr_rep2_ov	Difference in expression correlation of two repressors binding to overlapping sites with the target gene	
PercActrep_overlap	Percentage of overlapping sites shared by one activator and one repressor	
Avg_stractrep_ov	Average strength of regulation of activator and repressor binding to overlapping sites	
Df_stractrep_ov	Difference in strength of regulation of activator and repressor binding to overlapping sites	
Avg_corstr_actrep_ov	Average expression correlation of activator and repressor binding to overlapping sites with the target gene	
Df_corstr_actrep_ov	Difference in expression correlation of activator and repressor binding to overlapping sites with the target gene	
NumCoopTF_Yang2010	Number of cooperatively binding regulatory TFs from Yang et al., 2010 data	[17]
PercCoopTF_Yang2010	Percentage of cooperatively binding regulatory TFs from Yang et al., 2010 data	
PercOvNocpTF_Yang2010	Percentage of TFs showing binding site overlaps that are not cooperatively binding TFs as per Yang et al., 2010 data	
NumCoopTF_Chen2012	Number of cooperatively binding regulatory TFs from Chen et al., 2012 data	[18]
PercCoopTF_Chen2012	Percentage of cooperatively binding regulatory TFs from Chen et al., 2012 data	
PercOvNocpTF_Chen2012	Percentage of TFs showing binding site overlaps that are not cooperatively binding TFs as per Chen et al., 2010 data	
Pugh2004_SAGA_Dominance	Genes showing SAGA dominance in the promoter (Yes/No)	[19]
Pugh2004_TFIID_Dominance	Genes showing TFIID Dominance in the promoter (Yes/No)	
Pugh2004_SAGA_TFIID	Genes activated by both SAGA/TFIID complexes (Yes/No)	
Donczew2020_Coactivator_redundant_motif	Number of the coactivator redundant motif present in the promoter	[20]

Donczew2020_TFIID_dependent_motif	Number of the TFIID redundant motif present in the promoter	
TBP.NSMB2009_pol_II	Whether the promoter is a polII transcribed promoter (Yes/No)	[21]
TBP.NSMB2009_pol_III	Whether the promoter is a polIII transcribed promoter (Yes/No)	
TBP.NSMB2009_0	Ratio of inducible TBP expression level to constitutively expressed TBP level at time t=0	
TBP.NSMB2009_10	Ratio of inducible TBP expression level to constitutively expressed TBP level at time t=10 mins	
TBP.NSMB2009_20	Ratio of inducible TBP expression level to constitutively expressed TBP level at time t=20 mins	
TBP.NSMB2009_25	Ratio of inducible TBP expression level to constitutively expressed TBP level at time t=25 mins	
TBP.NSMB2009_30	Ratio of inducible TBP expression level to constitutively expressed TBP level at time t=30 mins	
TBP.NSMB2009_40	Ratio of inducible TBP expression level to constitutively expressed TBP level at time t=40 mins	
TBP.NSMB2009_60	Ratio of inducible TBP expression level to constitutively expressed TBP level at time t=60 mins	
TBP.NSMB2009_90	Ratio of inducible TBP expression level to constitutively expressed TBP level at time t=90 mins	
TBP.NSMB2009_TBP_occupancy	Overall TBP occupancy	
TBP.NSMB2009_TBP_turnover	TBP turnover rate	
HolstegeMSB2020_Abf1_NormBindingt0_Gene	Normalized binding levels of Abf1 in the coding region before nuclear depletion of Abf1	[22]
HolstegeMSB2020_Abf1_EstBindingt0_Gene	Estimate for binding levels of Abf1 in the coding region before nuclear depletion of Abf1	
HolstegeMSB2020_Abf1_Offrate_Gene	Abf1 binding offrate in the coding region	
HolstegeMSB2020_Abf1_MeanRestimeMins_Gene	Mean residence time of Abf1 in mins in the coding region	
HolstegeMSB2020_Abf1_NormBindingt0_Prom	Normalized binding levels of Abf1 in the promoter region before nuclear depletion of Abf1	
HolstegeMSB2020_Abf1_EstBindingt0_Prom	Estimate for binding levels of Abf1 in the promoter region before nuclear depletion of Abf1	
HolstegeMSB2020_Abf1_Offrate_Prom	Abf1 binding offrate in the promoter region	
HolstegeMSB2020_Abf1_MeanRestimeMins_Prom	Mean residence time of Abf1 in mins in the promoter region	
MolClutch_Nature2012_Rap1Residency_Gene	Residency of Rap1 in the coding region (in mins)	[23]
MolClutch_Nature2012_Rap1Occupancy_Gene	Occupancy of Rap1 in the coding region	
MolClutch_Nature2012_Rap1Residency_Prom	Residency of Rap1 in the promoter region (in mins)	
MolClutch_Nature2012_Rap1Occupancy_Prom	Occupancy of Rap1 in the promoter region	
GSE44200_2.5MNase_TBPocc_Gene	TBP occupancy in the coding region (results from 2.5min Mnase treatment)	[24]
GSE44200_2.5MNase_Mot1occ_Gene	Mot1 occupancy in the coding region (results from 2.5min Mnase treatment)	
GSE44200_2.5MNase_Mot1TBPrat_Gene	Ratio of Mot1 to TBP occupancy in the coding region (results from 2.5min Mnase treatment)	
GSE44200_2.5MNase_TBPocc_Prom	TBP occupancy in the promoter region (results from 2.5min Mnase treatment)	
GSE44200_2.5MNase_Mot1occ_Prom	Mot1 occupancy in the promoter region (results from 2.5min Mnase treatment)	
GSE44200_2.5MNase_Mot1TBPrat_Prom	Ratio of Mot1 to TBP occupancy in the promoter region (results from 2.5min Mnase treatment)	
GSE44200_10MNase_TBPocc_Gene	TBP occupancy in the coding region (results from 10min Mnase treatment)	
GSE44200_10MNase_Mot1occ_Gene	Mot1 occupancy in the coding region (results from 10min Mnase treatment)	

GSE44200_10MNase_Mot1TBPrat_Gene	Ratio of Mot1 to TBP occupancy in the coding region (results from 10min Mnase treatment)	
GSE44200_10MNase_TBPocc_Prom	TBP occupancy in the promoter region (results from 10min Mnase treatment)	
GSE44200_10MNase_Mot1occ_Prom	Mot1 occupancy in the promoter region (results from 10min Mnase treatment)	
GSE44200_10MNase_Mot1TBPrat_Prom	Ratio of Mot1 to TBP occupancy in the promoter region (results from 10min Mnase treatment)	
GSE59523_NucleosomeAsymmetry_Prom	Nucleosome assymetry (+1 or -1) in the promoter	[25]
YenEtAl_Cell2012_Arp5_Nuc	Whether the gene has Arp5 bound nucelosome (Yes/No)	[26]
YenEtAl_Cell2012_Ino80_Nuc	Whether the gene has Ino80 bound nucelosome (Yes/No)	
YenEtAl_Cell2012_Ioc3_Nuc	Whether the gene has Ioc3 bound nucelosome (Yes/No)	
YenEtAl_Cell2012_Ioc4_Nuc	Whether the gene has Ioc4 bound nucelosome (Yes/No)	
YenEtAl_Cell2012_Isw1_Nuc	Whether the gene has Isw1 bound nucelosome (Yes/No)	
YenEtAl_Cell2012_Isw2_Nuc	Whether the gene has Isw2 bound nucelosome (Yes/No)	
YenEtAl_Cell2012_Rsc8_Nuc	Whether the gene has Rsc8 bound nucelosome (Yes/No)	
YenEtAl_Cell2012_Snf2_Nuc	Whether the gene has Snf2 bound nucelosome (Yes/No)	
YenEtAl_Cell2012_Ioc4_terminalNuc	Whether the gene has Ioc4 bound terminal nucelosome (Yes/No)	
YenEtAl_Cell2012_Ioc3_terminalNuc	Whether the gene has Ioc3 bound terminal nucelosome (Yes/No)	
YenEtAl_Cell2012_Ino80_terminalNuc	Whether the gene has Ino80 bound terminal nucelosome (Yes/No)	
YenEtAl_Cell2012_Isw1_terminalNuc	Whether the gene has Isw1 bound terminal nucelosome (Yes/No)	
YenEtAl_Cell2012_Isw2_terminalNuc	Whether the gene has Isw2 bound terminal nucelosome (Yes/No)	
Intra_Gb_NumInt	Number of intra-chromosomal interactions in the genebody	[27]
Intra_Prom_NumInt	Number of intra-chromosomal interactions in the romoter	
Inter_Gb_NumInt	Number of inter-chromosomal interactions in the genebody	
Inter_Prom_NumInt	Number of inter-chromosomal interactions in the romoter	

References

1. Moskvina E, Schüller C, Maurer CT, Mager WH, Ruis H. A search in the genome of Saccharomyces cerevisiae for genes regulated via stress response elements. Yeast 1998; 14: 1041-1050.

2. Basehoar AD, Zanton SJ, Pugh BF. Identification and distinct regulation of yeast TATA box-containing genes. Cell 2004; 116:699-709

3. Lu Z, Lin Z. Pervasive and dynamic transcription initiation in Saccharomyces cerevisiae. Genome Res. 2019; 29: 1198-1210.

4. Tuller T, Carmi A, Vestsigian K, Navon S, Dorfan Y, Zaborske J, et al. An evolutionarily conserved mechanism for controlling the efficiency of protein translation. Cell 2010; 141: 344-354.

5. Oberbeckmann E, Wolff M, Krietenstein N, Heron M, Ellins JL, Schmid A, et al. Absolute nucleosome occupancy map for the Saccharomyces cerevisiae genome. Genome Res. 2019; 29: 1996-2009.

6. Pokholok DK, Harbison CT, Levine S, Cole M, Hannett NM, Lee TI, et al. Genome-wide map of nucleosome acetylation and methylation in yeast. Cell 2005; 122: 517-527.

7. Dion MF, Kaplan T, Kim M, Buratowski S, Friedman N, Rando OJ. Dynamics of replication-independent histone turnover in budding yeast. Science 2007; 315: 1405-8.

8. Sun M, Schwalb B, Schulz D, Pirkl N, Etzold S, Larivière L, et al. Comparative dynamic transcriptome analysis (cDTA) reveals mutual feedback between mRNA synthesis and degradation. Genome Res. 2012; 22: 1350-1359.

9. Kertesz M, Wan Y, Mazor E, Rinn JL, Nutter RC, Chang HY, et al. Genome-wide measurement of RNA secondary structure in yeast. Nature 2010; 467: 103-107.

10. Geisberg JV, Moqtaderi Z, Fan X, Ozsolak F, Struhl K. Global analysis of mRNA isoform half-lives reveals stabilizing and destabilizing elements in yeast. Cell 2014; 156: 812-824.

11. Belle A, Tanay A, Bitincka L, Shamir R, O'Shea EK. Quantification of protein half-lives in the budding yeast proteome. Proc Natl Acad Sci USA 2006; 103:13004-13009.

12. Ledesma L, Sandoval E, Cruz-Martínez U, Escalante AM, Mejía S, Moreno-Álvarez P, et al. YAAM: Yeast Amino Acid Modifications Database. Database (Oxford). 2018; 2018: bax099.

13. Teixeira MC, Monteiro PT, Palma M, Costa C, Godinho CP, Pais P, et al. YEASTRACT: an upgraded database for the analysis of transcription regulatory networks in Saccharomyces cerevisiae. Nucleic Acids Res. 2018; 46: D348-D353.

14. de Boer CG, Hughes TR. YeTFaSCo: a database of evaluated yeast transcription factor sequence specificities. Nucleic Acids Res. 2012; 40; D169-D179.

15. Newman JR, Ghaemmaghami S, Ihmels J, Breslow DK, Noble M, DeRisi JL, et al. Single-cell proteomic analysis of S. cerevisiae reveals the architecture of biological noise. Nature 2006; 441: 840-846.

16. Dhar R, Missarova AM, Lehner B, Carey LB. Single cell functional genomics reveals the importance of mitochondria in cell-to-cell phenotypic variation. Elife. 2019; 8: e38904.

17. Yang Y, Zhang Z, Li Y, Zhu XG, Liu Q. Identifying cooperative transcription factors by combining ChIP-chip data and knockout data. Cell Res. 2010; 20: 1276-1278.

18. Chen MJ, Chou LC, Hsieh TT, Lee DD, Liu KW, Yu CY, et al. De novo motif discovery facilitates identification of interactions between transcription factors in Saccharomyces cerevisiae. Bioinformatics 2012; 28:701-708.

19. Huisinga KL, Pugh BF. A genome-wide housekeeping role for TFIID and a highly regulated stress-related role for SAGA in Saccharomyces cerevisiae. Mol Cell. 2004; 13: 573-585.

20. Donczew R, Warfield L, Pacheco D, Erijman A, Hahn S. Two roles for the yeast transcription coactivator SAGA and a set of genes redundantly regulated by TFIID and SAGA. Elife 2020; 9: e50109.

21. van Werven FJ, van Teeffelen HA, Holstege FC, Timmers HT. Distinct promoter dynamics of the basal transcription factor TBP across the yeast genome. Nat Struct Mol Biol. 2009; 16:1043-1048.

22. de Jonge WJ, Brok M, Lijnzaad P, Kemmeren P, Holstege FC. Genome-wide off-rates reveal how DNA binding dynamics shape transcription factor function. Mol Syst Biol. 2020; 16: e9885.

23. Lickwar CR, Mueller F, Hanlon SE, McNally JG, Lieb JD. Genome-wide protein-DNA binding dynamics suggest a molecular clutch for transcription factor function. Nature 2012; 484: 251-255.

24. Zentner GE, Henikoff S. Mot1 redistributes TBP from TATA-containing to TATA-less promoters. Mol Cell Biol. 2013; 33: 4996-5004.

25. Ramachandran S, Zentner GE, Henikoff S. Asymmetric nucleosomes flank promoters in the budding yeast genome. Genome Res. 2015; 25: 381-390.

26. Yen K, Vinayachandran V, Batta K, Koerber RT, Pugh BF. Genome-wide nucleosome specificity and directionality of chromatin remodelers. Cell 2012; 149:1461-1473.

27. Duan Z, Andronescu M, Schutz K, McIlwain S, Kim YJ, Lee C, et al. A three-dimensional model of the yeast genome. Nature 2010; 465: 363-367.
